# Impact of the Fasting Plasma Glucose Titration Target on the Success of Basal Insulin Titration in Insulin-Naïve Patients with Type 2 Diabetes: A Systematic Analysis

**DOI:** 10.1155/2022/4758042

**Published:** 2022-07-30

**Authors:** Jannik Wolters, Dominik Wollenhaupt, Mirna Abd El Aziz, Michael A. Nauck

**Affiliations:** Diabetes Division, Katholisches Klinikum Bochum, St. Josef-Hospital, Ruhr-University Bochum, Bochum, Germany

## Abstract

**Background/Aim:**

We aimed to examine beneficial and adverse outcomes of basal insulin titration performed with different fasting plasma glucose (FPG) titration targets (TT).

**Methods:**

A PubMed literature search retrieved 43 reported prospective clinical trials introducing basal insulin in 17643 insulin-naïve patients with type 2 diabetes reporting fasting plasma glucose (FPG), HbA_1c_, target achievement, hypoglycemic events, and insulin doses. 61 individual study arms were grouped by fasting plasma glucose titration target (TT; 1: ≤5.0 mmol/l/90 mg/dl; 2: 5.01-5.6 mmol/l/90-100 mg/dl; and 3: ≥5.61 mmol/l/101 mg/dl). Weighted means and their standard deviations were calculated for baseline and end-of-treatment FPG (primary endpoint), HbA_1c_, target achievement, hypoglycemic events, insulin doses, and body weight gain and compared over a duration of 31 ± 10 weeks.

**Results:**

Achieved FPG and HbA_1c_ at the end of the study were significantly lower (by up to 0.8 mmol/l or 0.23%, respectively) with more ambitious TTs (*p* < 0.0001), leading to better HbA_1c_ target achievement with more ambitious TTs (by up to 14.6% for HbA_1c_ ≤ 6.5%), without increasing the risk for hypoglycemic episodes.

**Conclusions:**

Aiming for a lower FPG TT improves glycemic control without increasing the risk for hypoglycemia.

## 1. Introduction

Insulin therapy for type 2 diabetes usually is necessary after a longer duration of the disease due to its inherent tendency to progress in terms of insulin secretion capacity getting lower over time [1]. Guidelines, in principle, recommend various insulin regimens (once daily basal insulin plus oral glucose-lowering agents, premixed insulin preparations containing intermediate- or long-acting plus rapid-acting insulin preparations, mostly used with two injections per day, or intensified regimens, i.e., a combination of basal insulin once daily plus meal-related injections of a rapid-acting insulin preparation [2, 3]). In almost all patients, the initial insulin therapy will be basal insulin injected once daily, because such a regimen has a chance to take many patients to their individual glycemic targets with a relatively simple approach (at least compared to more advanced insulin regimens [2–5]).

When initiating basal insulin treatment in insulin-naïve patients, the dosage of insulin needs to be titrated individually, because the insulin need is highly variable between patients [6]. The immediate target for the titration process is the fasting plasma glucose concentration, which, together with overnight plasma glucose concentrations, usually defines the lowest plasma glucose concentrations of a typical 24 h period [4, 5, 7].

Several aspects of the titration process have varied between studies published on initiating basal insulin treatment in insulin-naïve patients: (a) various basal insulin preparations have been employed [5, 7–13]; (b) background oral glucose-lowering medications have differed by medication class (metformin [4, 7], sulfonylurea compounds [4, 14], inhibitors of dipeptidyl peptidase-4 (DPP-4) [8–11], sodium-glucose-co-transporter-2 (SGLT-2) [12], or thiazolidinediones [13]), i.e., by their mechanism of action and related adverse events (e.g., hypoglycemia with sulfonylureas [15, 16]); (c) titration is performed by the patient him or herself [7, 17, 18] or by health care professionals [5, 13]; and (d) guidance for the titration process has suggested different initial insulin doses [8, 17], different titration intervals (typically ranging from once every 3 days to once every 2 weeks or at the occasion of study visits) [7, 14, 19], and different algorithms varying with respect to their “stringency” (i.e., by how much the insulin dose is increased in case of hyperglycemia) [20, 21]. Last, not least, there is quite some variation in reported fasting plasma glucose titration targets, overall ranging from ≤4.9 [14] to ≤6.2 mmol/l [22]. It can be assumed that the titration target has a prominent role in determining the success of basal insulin therapy, especially since there is some evidence that basal insulin therapy has the potential to improve meal-related insulin secretion and to lower postmeal glycemic excursions, if it only supports fasting glucose concentrations near the normal fasting range [23, 24]. However, there is no generally agreed fasting plasma glucose titration target, and it remains unknown whether more ambitious fasting plasma glucose titration targets are associated with better glycemic control or whether they rather lead to problems related to higher insulin doses, an increased prevalence and/or incidence of hypoglycemia, or weight gain.

It is our impression that the majority of clinical trials has mainly focused on the comparison of different insulin preparations [5, 7–9, 11–13, 19, 21, 25–28]. Technical aspects regarding the optimization of the insulin titration process and the eventual results have often not been examined. In the present systematic analysis, we aim to assess differences between categories of fasting plasma glucose titration targets with respect to their success (fasting plasma glucose and HbA_1c_ concentrations and target achievements and concerning associated risks (hypoglycemia, weight gain).

## 2. Patients and Methods

### 2.1. Search Strategy and Study Selection

For the present analysis, articles reporting prospective, randomized, blinded, or open-label clinical trials of initiating basal insulin treatment in insulin-naïve type 2 diabetic patients on a background of a well-defined therapy with single or combined oral glucose-lowering agents were identified through a systematic PubMed search. The search terms are displayed in Supplementary Table S1. We searched for prospective, randomized, clinical trials published between 1999 and October 2020 providing details on the basal insulin titration process like (a) insulin preparations used; (b) background oral glucose-lowering medications (at least by class); (c) person performing the titration; (d) initial insulin doses; (e) titration intervals (categorized as daily, every 3 days or twice a week, and weekly or in association with study visits (including telephone contacts) only) or the number of titration opportunities (multiplying the occasions per week with the total study duration); (f) “stringency” of the titration algorithm (steepness of the relationship between categories of hyperglycemia and the proposed increment in basal insulin dose); and (g) fasting plasma glucose titration targets. Additional inclusion criteria were (h) study duration ≥24 weeks, (i) a minimum number of 50 patients per study arm, and (k) report of essential information regarding baseline characteristics (age, sex, duration of diabetes, body weight and body mass index, fasting plasma glucose, and HbA_1c_) and relevant outcomes at the completion of the study (fasting plasma glucose, HbA_1c_, and HbA_1c_ target achievement < 7.0% (<53.0 mmol/mol) and ≤6.5 (47.5 mmol/mol), insulin dose after titration (per day and/or per kg body weight and day), change in body weight, and the proportion of patients reporting any symptomatic or severe hypoglycemia. Exclusion criteria were publications reporting cross-over studies, concerning other types of diabetes, reporting results concerning specific ethnic groups other than Caucasian or internationally mixed populations only, studies allowing concomitant use of GLP-1 receptor agonists (exception: <5.0% of the study population as a consequence of protocol violations, overall), studies with >10% patients with preexisting basal insulin therapy, and studies reporting >5% of patients treated with rapid-acting insulin preparations as part of the rescue strategy. Of 1060 records identified initially, 43 publications representing 61 study arms could be used. Exclusion criteria are described in Supplementary Figure S1 according to the PRISMA statement [29]. We registered our protocol with PROSPERO (https://www.crd.york.ac.uk/prospero/; identification no. CRD42019134821).

### 2.2. Design of the Analysis

Individual study arms were analyzed if they fulfilled the inclusion and exclusion criteria. These study arms were grouped by the fasting plasma glucose titration targets reported. Based on the distribution of fasting plasma glucose titration targets employed in these study arms, they were grouped into fasting plasma glucose titration targets 1 (≤5.00 mmol/l), 2 (5.01-5.60 mmol/l), and 3 (≥5.61 mmol/l) and compared. Since the main focus of our analysis is the achievement of ambitious FPG and HbA_1c_ targets, our main endpoint was FPG after completing the titration process (the immediate consequence of basal insulin titration), and our secondary endpoints included HbA_1c_ concentrations and target achievement after titration.

### 2.3. Quality Assessment

Study quality was assessed applying the Jadad score [30] and the Risk of Bias tool (https://www.riskofbias.info/) [31]. All publications turned out to be suitable for our analysis.

### 2.4. Data Extraction

Relevant data were extracted into prestructured paper forms listing variables of interest. Data were extracted by JW and DW. In case of questions or discrepancies, MAN was consulted. In case of differences that could not be resolved, MAN had the final decision.

### 2.5. Data Synthesis and Systematic Analysis

Fasting plasma glucose achieved after basal insulin titration was the primary endpoint. Secondary endpoints were HbA_1c_ after basal insulin titration, fasting plasma glucose (as defined in individual study arms), and HbA_1c_ target achievements (<7.0% (53.0 mmol/mol) and ≤6.5 (≤47.5 mmol/mol)). Safety endpoints were the proportion of patients reporting any symptomatic or severe hypoglycemia. Exploratory endpoints were the insulin dose after titration (per day and/or per kg body weight and day), insulin dose, and body weight change vs. baseline (study end vs. baseline). All endpoints were compared between pooled study arms belonging to the same fasting plasma glucose titration target category. Within-group weighted means and pooled standard deviations were calculated using established equations assuming normal distribution of data. Heterogeneity was reported as *Q* value, the associated *p* value, and *I*^2^.

### 2.6. Exploratory Analyses

Along the same lines, we also examined potential differences in the same outcomes by the number of occasions for titration (two groups: 10-30 vs. 31-72 occasions, based on the frequency of titration and the total study durations) and by categories of “stringency” of the titration algorithm (one-step algorithm requesting the same increment in insulin doses irrespective of the degree of fasting hyperglycemia) and stepped algorithm with a weak (maximum basal insulin dose increment 2-4 U in the highest category of hyperglycemia mentioned) or strong (maximum basal insulin dose increment ≥ 5 U in the highest category of hyperglycemia mentioned) degree of stringency. A fourth category was titration at the discretion of the investigator (without presenting any detailed guidance).

### 2.7. Regression Analyses

A linear regression analysis was performed relating fasting plasma glucose and HbA_1c_ achieved after basal insulin titration. The regression equation *r*^2^ and the respective *p* values are reported for this association.

### 2.8. Estimation of Fasting Plasma Glucose Target Achievement

We analyzed the proportion of patients reaching their individual fasting plasma glucose titration targets from mean values ± standard deviations, assuming a normal distribution, using the function “normal distribution” implemented in Microsoft Excel (version 16.0.13929.20206).

### 2.9. Statistical Analysis

Baseline patient characteristics and results at the end of the study are reported as means ± standard deviation (SD) or proportions (percentages). 95% confidence intervals were derived from standard deviations and the number of patients in the respective category. Weighted mean values and pooled standard deviations for all studies belonging to one subgroup or all studies pooled were calculated using standard equations. For continuous variables, *p* values for significant differences were calculated by analysis of variance assuming that standard deviations were different (Brown-Forsythe and Welch method) for comparing 3 groups (fasting plasma glucose titration target categories) with post hoc comparisons between individual groups by the Games-Howell test [20]. For continuous variables, a *χ*^2^ test for larger than 2 × 2 contingency tables and Fisher's exact test for 2 × 2 contingency tables (e.g., post hoc tests to identify significant differences between specific titration targets) were used. No adjustment was made for multiple comparisons. Exact *p* values are presented. *p* values < 0.05 were taken to indicate significant differences.

### 2.10. Sensitivity Analysis

Since the studies analyzed used various basal insulin preparations, but 40 out of 61 study arms employed insulin glargine U-100, we repeated our primary analysis with study arms employing insulin glargine U-100 only. In a similar way, we repeated the analysis for (the majority of) studies allowing sulfonylureas.

## 3. Results

### 3.1. Selection of Publications

The search terms for the retrieval of publications and the selection of study arms for the present analysis are illustrated in Supplementary Table S1 and Supplementary Figure S1. Overall, 61 arms from 43 publications could be used for the present analysis, representing 17643 patients divided among 3 categories of different fasting plasma glucose titration targets used with basal insulin and concomitant oral glucose-lowering medications.

### 3.2. Quality Assessment

The quality of the studies assessed by the Jadad score [30] (Supplementary Table S2) and the Cochrane Collection Risk of Bias tool [31] (Supplementary Figure S2) was found to be sufficient for the inclusion of all retrieved publications and relevant study arms.

### 3.3. Baseline Characteristics

Baseline patient characteristics of all studies analyzed, summarized by fasting plasma glucose titration target, are shown in Table 1 and Supplementary Tables S3 and S4. Patient age was equally distributed across fasting plasma glucose titration targets, while the proportion of females was lower in patients belonging to fasting plasma glucose titration target 1. Study duration was significantly shorter going from fasting plasma glucose titration targets 1 to 3. Regarding concomitant use of oral glucose-lowering medications, studies summarized as fasting plasma glucose titration target 1 had a lower proportion treated with sulfonylureas/meglitinides (Table 2). There were subtle differences in body mass index and body weight, and baseline HbA_1c_ and fasting plasma glucose were lowest for fasting plasma glucose titration target 1, intermediate for fasting plasma glucose titration target 2, and highest for fasting plasma glucose titration target 3 (Table 1). However, mean differences maximally were 0.5% (5.5 mmol/mol) for HbA_1c_ and 1.6 mmol/l for fasting plasma glucose (Table 1).

### 3.4. Study Characteristics including Differences in the Basal Insulin Titration Strategy

Further study protocol details are shown in Table 2. Aspects of the titration algorithm were not significantly different for the titration interval, the person performing the titration, the starting dose of basal insulin, and the “stringency” of the titration algorithm (Table 2). By evaluating the insulin preparations used, there was a significant difference between fasting plasma glucose titration targets, with a relatively higher use of insulin degludec in studies summarized as fasting plasma glucose titration target 1 and relatively more use of insulin glargine U-100 in studies summarized as fasting plasma glucose titration target 2 (Table 2).

### 3.5. Primary Endpoint

**Figure 1:**
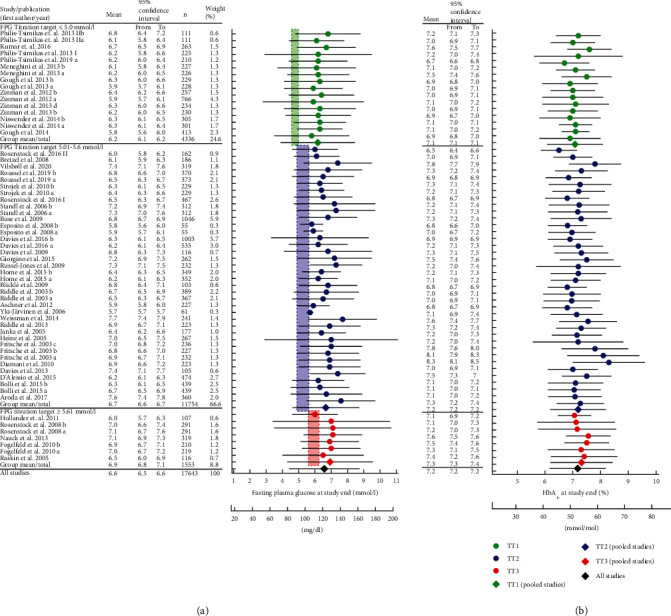
Forrest plot of fasting plasma glucose (a) and HbA_1c_ (b) concentrations achieved after basal insulin titration according to categories of fasting plasma glucose titration target (TT): 1 (≤5.0, green symbols), 2 (5.01-5.60 mmol/l, blue symbols), and 3 (≥5.60 mmol/l; red symbols). Individual studies are shown with filled circles, and pooled results reflecting the three categories of fasting plasma glucose targets are shown as diamonds. Means ± standard deviation. The range of fasting plasma glucose titration targets for the three categories is shown as a colored shaded area.

Fasting plasma glucose at the end of the study, i.e., as the result of the basal insulin titration process, was lowest with fasting plasma glucose titration target 1, intermediate with fasting plasma glucose titration target 2, and highest with fasting plasma glucose titration target 3. Differences were significant between all three categories of fasting plasma glucose titration targets (Table 3, Figure 1). The maximum difference between mean results for fasting plasma glucose titration targets 1 and 3 amounted to 0.8 mmol/l (Table 3).

### 3.6. Secondary Endpoints

The distribution of HbA_1c_ concentrations at the end of the study followed the pattern seen with fasting plasma glucose (Table 3, Figure 1). Differences were significant between all three categories of fasting plasma glucose titration targets. The maximum difference between mean results for fasting plasma glucose titration targets 1 and 3 amounted to 0.2% (2.2 mmol/mol; Table 3). There was a highly significant correlation of fasting plasma glucose and HbA_1c_ (Supplementary Figure S3), and values at baseline fell onto the same regression line as values at the end of the study.

HbA_1c_ target achievement reached a higher proportion reaching <7.0 and ≤6.5% (<53.0 and ≤47.5 mmol/mol) going from fasting plasma glucose titration targets 3 to 1 (*p* < 0.0001), maximally amounting to differences by 13.0 and 14.6% between fasting plasma glucose titration targets 1 and 3 (Table 3).

### 3.7. Exploratory Endpoints

Insulin doses after titration were highest with fasting plasma glucose titration target 1, with significant differences between all fasting plasma glucose titration targets (Table 3, *p* < 0.0001; Supplementary Table S5).

Body weight tended to increase by approximately 1.5 kg in all groups and was lowest with fasting plasma glucose titration target 1, i.e., the most ambitious fasting plasma glucose titration target (Table 3).

### 3.8. Safety Endpoint: Hypoglycemia

The proportion of patients reporting any symptomatic hypoglycemia decreased gradually going from fasting plasma glucose titration targets 3 to 1 (*p* < 0.0001). Analyzing studies by their use of sulfonylureas led to a lower risk for hypoglycemia in those study arms not employing sulfonylureas but confirmed the pattern seen for the overall analysis. However, in studies not using sulfonylureas, the proportion reporting hypoglycemia increased significantly comparing fasting plasma glucose titration targets 3 and 1 (Table 3).

The proportion of patients reporting nocturnal hypoglycemia, likewise, did not increase with more ambitious fasting plasma glucose titration targets (Table 3). These results were reported in 21 out of 43 studies (34 of 62 study arms) and indicated a lower proportion of patients reporting nocturnal hypoglycemia compared to those experiencing any hypoglycemic episode.

A very similar pattern was seen for the proportion of patients experiencing severe hypoglycemia, which decreased going from fasting plasma glucose titration targets 3 to 2 to 1 (all differences were significant; Table 3).

### 3.9. Fasting Plasma Glucose Titration Target Achievement

Looking at fasting plasma glucose target achievement (against the individually defined fasting plasma titration targets), it was reached in only 29.1% (95% confidence interval, 28.5 to 29.8%) of the patients overall, with little differences when differentiating by the fasting plasma glucose titration target category. It appeared slightly more successful to achieve less ambitious fasting plasma glucose titration targets (Supplementary Figure S4).

### 3.10. Sensitivity Analysis

Repeating the primary analysis with studies employing insulin glargine U-100 only fully confirmed the analysis including all insulin preparations, with the same significant differences regarding end-of-titration FPG and HbA_1c_ (details not shown). Likewise, when only studies allowing sulfonylureas were analyzed, the results regarding FPG fully confirmed our main analysis (including significant differences between all three FPG titration targets), while only a similar trend was observed regarding end-of-study HbA_1c_.

### 3.11. Exploratory Analyses

Regarding other aspects of the titration algorithms (Table 2), additional analyses indicated that there were significant influences of the number of opportunities to titrate basal insulin (as outlined in the respective protocols, partially depending on study duration), with more opportunities being associated with better glycemic results (Supplementary Tables 6 and 7): with 31-72 opportunities (vs. 10-30) to titrate, HbA_1c_ was better by 0.15% and HbA_1c_ target achievements were higher by 4.1% (target < 7.0%) and 6.0% (≤6.5%), respectively. Insulin doses achieved after titration were slightly but significantly increased with a greater number of titration opportunities, and the proportion of patients reporting hypoglycemic episodes increased, while the change in body weight was similar (Supplementary Tables S6 and S7). It is remarkable that regarding the “stringency” of titration algorithms, the best glycemic outcomes were observed when leaving the titration to the discretion of the study team. Between algorithm-based titration protocols, a higher degree of “stringency” (requesting greater increments in insulin doses per titration step with higher degrees of hyperglycemia) did not improve outcomes (Supplementary Tables S8 and S9).

## 4. Discussion

The main finding of the present analysis is that aiming for more ambitious (lower) fasting plasma glucose titration targets leads to slightly but significantly better glycemic control as measured by the resulting fasting plasma glucose and HbA_1c_ concentrations after titrating basal insulin in insulin-naïve patients with type 2 diabetes on a background of oral glucose-lowering medications (Table 3, Figure 1). Furthermore, there was no obvious risk associated with these more ambitious titration targets, since the proportion of patients reporting any symptomatic or even severe hypoglycemia and the weight gain observed with initiating basal insulin therapy were not higher aiming for lower fasting plasma glucose titration targets.

These results should encourage the recommendation to aim for a plasma glucose titration target similar to our category 1, which ranged from 4.9 to 5.0 mmol/l regarding the upper range of the targeted fasting plasma glucose concentrations. It is not known whether even lower targets will help achieve better glycemic control, or whether doing so will provoke unwanted consequences regarding hypoglycemia and body weight gain.

Aiming for such stringent basal insulin titration will most likely help exploit the therapeutic potential of basal insulin therapy, especially with the observation in mind that the achievement of fasting plasma glucose concentrations near the normal fasting range will allow improvements in acute insulin secretory responses with intravenous and oral meal stimuli [23, 32] and has a chance to also affect postprandial glycemic excursions [33], besides lowering fasting plasma glucose alone (which remains the primary mode of action of basal insulin).

While demonstrating better glycemic control aiming for more ambitious fasting plasma glucose titration targets encourages the use of such targets as component of the basal insulin titration strategy, implemented in dedicated titration algorithms, it is disappointing to see the degree of individual fasting plasma glucose target achievement (Supplementary Figure S4). Our study does not provide a clue as to why the degree of fasting plasma glucose target achievement is relatively low. We only included studies with a minimum duration of 24 weeks, in order to allow enough time and sufficient occasions to follow the titration algorithms (Table 2). Several publications used for the present systematic analysis present time courses for the rise in insulin doses used over time, which usually display a plateau during the latter part of the titration period [7, 8, 13, 14, 17, 20, 34–36]. This seems to indicate that it was not only for the lack of time or opportunities (Table 2) that insulin doses were not increased even without having achieved the algorithm-derived fasting plasma glucose titration target. The question arises whether the low fasting plasma glucose target achievement is rather causally related to the significant risk for any symptomatic or even severe hypoglycemia associated with basal insulin treatment in the studies analyzed in the present study (Table 3). Assume that the fasting plasma glucose should be at the lower end of the range of plasma glucose concentrations that can be measured during a 24 h period, since meals will rather increase glycemia during the day. This view would leave the overnight fasting period as the vulnerable period for the occurrence of hypoglycemic episodes. However, the risk for nocturnal hypoglycemia did not increase (but rather decreased) with more ambitious fasting plasma glucose titration targets (Table 3). We suggest that it might be helpful to employ continuous glucose monitoring in future studies aiming at the optimization of basal insulin titration algorithms, which should help to identify the role of low plasma glucose concentrations or hypoglycemia as a barrier to increasing insulin doses.

An alternative explanation would be day-to-day variations in fasting glucose, most likely due to variable absorption of basal insulin preparations [37, 38], which could explain occasional low plasma glucose concentrations even when the fasting plasma glucose concentration reported at the end of the study was in or above the target range (Supplementary Figure S4).

It should be noted that the three subgroups, defined by their fasting plasma glucose titration targets, did not only differ in this respect, but there were some imbalances in baseline characteristics (Table 1) and details of the basal insulin titration procedure (Table 2), which, in addition to the primary classification, might have affected the results. We consider it unlikely that a shorter trial duration (Table 1) should be associated with better glycemic results after titration, unless one assumes a decreasing effectiveness during the latter part of the study period with longer duration of the study, e.g., due to diabetes progression or waning adherence to lifestyle measures reinforced as part of recruitment into the clinical trials. The time course of fasting plasma glucose and HbA_1c_ reported in some of the studies does not suggest that this is of major influence [5, 8, 19, 29, 34, 39, 40]. The preferential use of insulin degludec in 37.8% of patients in the fasting plasma glucose titration target category 1 vs. none in the other groups might have influenced the results. Differences in the use of insulin preparations have been addressed with a sensitivity analysis focusing on all studies employing insulin glargine U-100, which fully confirmed the findings of the overall study. Therefore, we cannot find hints that an imbalance in the use of certain insulin preparations introduced a major bias.

Another point is the relatively low proportion of patients treated with sulfonylureas in studies with the most ambitious FPG titration target (5.2 vs. 63.5 or 68.3% with fasting plasma glucose titration targets 1, 2, and 3; *p* = 0.020). In an attempt to judge the influence of differences in the use of sulfonylureas between the three FPG titration targets, we analyzed studies allowing sulfonylureas only as another sensitivity analysis. Regarding end-of-treatment FPG, these results fully confirm the conclusions from the main analysis (details not shown). However, end-of-trial HbA_1c_ concentrations only demonstrated minor differences, perhaps related to a preferential reduction of postprandial plasma glucose concentrations induced by sulfonylureas. We are aware that the better results with more ambitious fasting plasma glucose titration targets may in part reflect the more favorable baseline conditions (fasting plasma glucose and HbA_1c_; Table 1) in the groups finally achieving better glycemic control at the end of the study. However, the long duration of the studies should have allowed study populations with less favorable baseline conditions to catch up with those starting with lower baseline fasting plasma glucose and/or HbA_1c_. There was, however, no difference in diabetes duration, which may be a better parameter predicting difficulties in achieving glycemic targets (Table 1), and body mass index was even highest in fasting plasma glucose titration target category 1.

The higher proportion of patients reporting hypoglycemic episodes in the category with more opportunities for titration may in part reflect the longer duration of the trials in this category. The superiority of titration “at the discretion of the investigator” vs. algorithm-based titration regimens (Supplementary Tables S8 and S9) points to a potential for greatly improving available algorithms with the aim of better target achievement.

Limitations of the present study are the mentioned imbalances in baseline characteristics (Table 1) and details of the titration protocols (Table 2), the influence of which on the main study results remains uncertain (as discussed above), and the many aspects characterizing details of the basal insulin titration process which have not been reported in the publications used for the present systematic analysis still might be confounders with a hidden influence on the results. It is well known that higher baseline HbA_1c_ values reduce the probability of achieving ambitious FPG and HbA_1c_ targets [15]. The better results in the studies with the most ambitious FPG titration target may in part be the result of their lower baseline FPG and HbA_1c_ concentrations (Table 1). Strengths of the present analysis are the systematic nature, the clear definition of inclusion and exclusion criteria, and the large number of studies (as well as study arms and patient numbers) analyzed.

In conclusion, clinical trials reporting basal insulin titration in hitherto insulin-naïve patients with type 2 diabetes indicate better glycemic results (fasting plasma glucose and HbA_1c_ at the end of the study) with more ambitious fasting plasma glucose titration targets, without showing associated risks or worse results in terms of safety consequences (hypoglycemia and weight gain). However, despite the degree of significance, the resulting differences were small. The overall fasting plasma glucose target achievement was low, and further studies are needed to identify barriers to more stringent basal insulin titration, e.g., day-to-day fluctuations in insulin absorption and fasting plasma glucose and the associated risk for nocturnal hypoglycemic episodes.

## Figures and Tables

**Table 1:** Baseline characteristics of insulin-naïve type 2 diabetic patients initiating basal insulin therapy by titration target.

Outcome parameter	Unit	Titration target 1: ≤5.0 mmol/l	Titration target 2: 5.01-5.6 mmol/l	Titration target 3: ≥5.61 mmol/l	Overall significance (*p* value)
Age	Years	58 ± 10	58 ± 9	58 ± 10	>0.99
Sex	Female/male (% female)	1877/2459 (43.3)	5446/6308 (46.3)^∗^	711/842 (45.8)	0.0026
BMI	kg/m^2^	31.6 ± 5.1	31.4 ± 5.2	31.0 ± 5.0^∗^^,†^	0.0003
Body weight	kg	90.1 ± 18.2	88.6 ± 18.5^∗^	88.6 ± 18.2^∗^	<0.0001
Duration of diabetes	Years	9 ± 6	9 ± 8	9 ± 6	>0.99
HbA_1c_	%	8.2 ± 0.8	8.6 ± 0.9^∗^	8.7 ± 0.8^∗^^,†^	<0.0001
	mmol/mol	66.6 ± 9.3	70.3 ± 10.3	71.7 ± 9.2	
Fasting plasma glucose	mmol/l	9.5 ± 2.5	10.2 ± 2.8^∗^	11.1 ± 4.4^∗^^,†^	< 0.0001
Study duration	Weeks	29 ± 9	31 ± 10^∗^	33 ± 13^∗^^,†^	<0.0001

Weighted group means ± common standard deviations (continuous variables) or number fulfilling/not fulfilling the criterion and the proportion (percentage) fulfilling the criterion in question (categorical variables). Statistical significance was assessed using one-way ANOVA (Welch's test) for continuous variables and the *χ*^2^ test for larger than 2 × 2 contingency tables and Fisher's exact test for 2 × 2 contingency tables (e.g., post hoc tests to identify significant differences between specific titration targets; exact *p* values are presented. ^∗^Significantly different (*p* < 0.05) vs. FPG titration target 1 (≤5.0 mmol/l). ^†^Significantly different (*p* < 0.05) vs. FPG titration target 2 (5.01-5.60 mmol/l).

**Table 2:** Study characteristics by titration target for publications included in the present systematic analysis of insulin-naïve type 2 diabetic patients initiating basal insulin therapy in combination with oral glucose-lowering drugs. Displayed are the numbers of study arms or patients and the proportion (percentage) in this particular subgroup defined by the titration target.

FPG titration target	FPG titration target, ≤5.0 mmol/l (mean, 4.96 mmol/l)	FPG titration target, 5.01-5.6 mmol/l (mean, 5.56 mmol/l)	FPG titration target, ≥5.61 mmol/l (mean, 6.01 mmol/l)	Overall significance
Basal insulin				0.0001
Neutral Protamine Hagedorn (NPH)	0 (0.0)	3 (7.9)	0 (0.0)	
Glargine U-100	7 (43.8)	30 (78.9)	3 (42.9)	
Glargine U-300	0 (0.0)	1 (2.6)	0 (0.0)	
Detemir	3 (18.8)	1 (2.6)	3 (42.9)	
Degludec	6 (37.8)	0 (0.0)	0 (0.0)	
Peglispro	0 (0.0)	1 (2.6)	0 (0.0)	
Insulin lispro protamine	0 (0.0)	2 (5.3)	1 (14.3)	
Oral glucose-lowering medication^∗^				0.020
Metformin	17 (99.5)	32 (84.3)	8 (94.5)	
Sulfonylurea compounds/meglitinides	1 (5.2)	23 (63.5)	5 (68.3)	
Thiazolidinediones	4 (6.8)	10 (6.2)	4 (8.7)	
DPP-4 inhibitors	6 (7.0)	3 (5.2)	1 (4.2)	
SGLT-2 inhibitors	1 (4.3)	0 (0.0)	0 (0.0)	
Titration interval				0.82
Twice a week/every three days	5 (31.3)	13 (34.1)	2 (28.6)	
Weekly	9 (56.3)	18 (47.4)	5 (68.3)	
During official study visits	2 (12.4)	5 (13.2)	0 (0.0)	
Other	0 (0.0)	1 (2.6)	0 (0.0)	
Not reported	0 (0.0)	1 (2.6)	0 (0.0)	
Person performing titration				0.24
Investigator	11 (68.8)	27 (71.1)	7 (100.0)	
Participant	5 (31.3)	11 (28.9)	0 (0.0)	
Patient education in association with recruitment into the study	7 (43.8)	15 (39.5)	3 (42.9)	0.95
Starting dose of basal insulin				0.08
<10 U/d	0 (0.0)	5 (13.2)	1 (14.3)	
10 U/d	13 (81.3)	20 (52.6)	4 (57.1)	
>10 U/d	0 (0.0)	11 (28.9)	2 (28.6)	
Not reported	3 (18.8)	2 (5.3)	0 (0.0)	
Titration stringency/algorithm				0.15
One-step algorithm	4 (25.0)	2 (5.3)	2 (28.6)	
Stepped algorithm, weak (2-4 IU)	1 (1.6)	12 (31.6)	1 (14.3)	
Stepped algorithm, strong (max. 5-8 IU)	9 (56.3)	20 (52.6)	4 (57.1)	
At the discretion of the investigator	2 (12.5)	2 (5.3)	0 (0.0)	
Occasions of titration				0.54
10-30	9 (56.3)	16 (42.1)	5 (71.4)	
31-72	5 (31.3)	18 (47.4)	2 (28.6)	
Not reported	2 (12.5)	4 (10.5)	0 (0.0)	

^∗^Presented are the number of study arms in which this class of oral glucose-lowering medications was used and the percentage of patients receiving this type of medication across all these study arms.

**Table 3:** Results achieved in clinical trials of basal insulin titration in insulin-naïve patients with type 2 diabetes using various basal insulin preparations in addition to well-defined single or combined oral glucose-lowering agents, trying to aim at different fasting plasma glucose titration targets.

		Overall comparison across all titration targets				Post hoc comparison of specific titration targets		
Outcome parameter	Unit	Titration target 1: ≤5.0 mmol/l	Titration target 2: 5.01-5.6 mmol/l	Titration target 3: ≥5.61 mmol/l	Overall significance (*p* value)	*Δ* titration target, ≤5.0 vs. 5.01-5.6 mmol/l	*Δ* titration target, ≤5.0 vs. ≥5.61 mmol/l	*Δ* titration target, 5.01-5.6 vs. ≥5.61 mmol/l
Fasting plasma glucose	mmol/l	6.17 (6.11; 6.24)	6.67 (6.63; 6.71)^∗^	6.92 (6.79; 7.06)^∗^^,†^	< 0.0001	0.50 (0.40; 0.59)^‡^	0.75 (0.58; 0.93)^‡^	0.25 (0.09; 0.42)^‡^
HbA_1c_	% mmol/mol	7.08 (7.05; 7.11) 53.9 (53.6; 54.2)	7.19 (7.18; 7.21)∗ 55.1 (55.0; 55.3)	7.32 (7.26; 7.37)^∗^^,†^ 56.5 (55.8; 57.0)	<0.0001	0.11 (0.07; 0.15)^‡^ 1.2 (0.8; 1.6)	0.23 (0.16; 0.31)^‡^ 2.5 (1.7; 3.4)	0.12 (0.05; 0.19)^‡^ 1.3 (0.5; 2.1)
HbA_1c_ < 7% (<53.0 mmol/mol)	Yes/no (% yes)	2248/2088 (51.9)	5116/5661 (47.5)^∗^	603/950 (38.8)^∗^^,†^	<0.0001	-4.4 (-6.1; -2.6)^‡^	-13.0 (-15.9; -10.1)^‡^	-8.6 (-11.3; -6.0)^‡^
HbA_1c_ ≤ 6.5% (<47.5 mmol/mol)	Yes/no (% yes)	434/867 (33.4)	1340/3838 (25.9)^∗^	182/789 (18.7)^∗^^,†^	<0.0001	-7.5 (-10.4; -4.7)^‡^	-14.6 (-18.2; -11.0)^‡^	-7.1 (-9.8; -4.3)^‡^
Daily insulin dose	U/d	62 (61; 63)	42 (41; 42)^∗^	47 (46; 49)^∗^^,†^	<0.0001	-20 (-22; -19)^‡^	-15 (-17; -12)^‡^	5 (4; 7)^‡^
Daily insulin dose	U/kg/d	0.66 (0.65; 0.67)	0.46 (0.45; 0.47)^∗^	0.53 (0.51; 0.54)^∗^^,†^	<0.0001	-0.20 (-0.22; -0.19)^‡^	-0.13 (-0.16; -0.11)^∗∗∗^	0.07 (0.05; 0.09)^‡^
Hypoglycemia	Yes/no (% yes)	1700/2413 (41.3)	5744/4987 (53.5)^∗^	767/786 (49.4)^∗^^,†^	<0.0001	12.2 (10.4; 14.0)^‡^	8.1 (5.1; 11.0)^‡^	-4.1 (-6.8; -1.5)^‡^
Studies allowing sulfonylureas/meglitinides	Yes/no (% yes)	96/129 (42.7)	4372/3412 (56.2)^∗^	661/350 (65.4)^∗^^,†^	<0.0001	13.5 (6.9; 20.0)^‡^	22.7 (15.6; 30.1)^‡^	9.2 (6.0; 12.5)^‡^
Studies not allowing sulfonylureas/meglitinides	Yes/no (% yes)	1604/2284 (41.3)	1372/1575 (46.6)^∗^	106/436 (19.6)^∗^^,†^	<0.0001	5.3 (2.9; 7.7)^‡^	-21.7 (-25.3; -17.7)^‡^	-27.0 (-30.1; -22.9)^‡^
Severe hypoglycemia	Yes/no (% yes)	18/4108 (0.4)	137/11431 (1.2)^∗^	21/1425 (1.5)^∗^^,†^	<0.0001	0.8 (0.5; 1.1)^‡^	1.1 (0.2; 1.6)^‡^	0.3 (-0.6; 0.8)
Nocturnal hypoglycemia	Yes/no (% yes)	542/2702 (16.7)	1892/3803 (33.2)	365/491 (42.6)	<0.0001	16.5 (14.7; 18.3)^‡^	25.9 (22.3; 29.5)^‡^	9.4 (5.8; 13.0)^‡^
Body weight change from baseline	kg	1.3 (1.2; 1.5)	1.9 (1.8; 1.9)^∗^	1.7 (1.5; 2.0)^∗^	<0.0001	0.6 (0.3; 0.7)^‡^	0.4 (0.1; 0.7)^‡^	-0.2 (-0.2; 0.4)

Continuous variables are presented as mean and their 95% confidence intervals, and categorical variables are presented as number fulfilling/not fulfilling the criterion and the proportion (percentage) fulfilling the criterion in question. Statistical significance was assessed using one-way ANOVA (Welch's test) for continuous variables and the *χ*^2^ test for larger than 2 × 2 contingency tables and Fisher's exact test for 2 × 2 contingency tables (e.g., post hoc tests to identify significant differences between specific titration targets), including the “attributable difference” expressed as a percentage and its 95% confidence interval. For overall comparisons, exact *p* values are presented. ^∗^Significantly different (*p* < 0.05) vs. FPG titration target 1 (≤5.0 mmol/l). ^†^Significantly different (*p* < 0.05) vs. FPG titration target 2 (5.01-5.6 mmol/l). For comparison of individual titration targets, significance is indicated by ‡.

## Data Availability

This manuscript analyses published data. There is no original dataset associated with this manuscript that we could share.

## Supplementary Materials

Supplementary Materials Supplementary Table S1: detailed presentation of search terms used for the PubMed search to identify studies included in the present systematic analysis investigating the impact of the fasting plasma glucose titration target on the success of basal insulin titration in insulin-naïve patients with type 2 diabetes. Supplementary Figure S1: flow diagram (according to the PRISMA statement) of the search process, selection, and exclusion of publications, which were identified through a systematic PubMed search and included in the present analysis. Data extraction form. Supplementary Table S2: the Jadad score estimating the study quality of the randomized controlled trials included in the present analysis. Supplementary Figure S2: risk of bias as assessed with the “Risk of Bias tool” 2.0 proposed by the Cochrane Collection for all studies included in the present systematic analysis. Supplementary Table S3: patients' baseline characteristics (age, sex, BMI, and body weight) for study arms in the present analysis. Supplementary Table S4: patients' baseline characteristics (duration of diabetes, fasting plasma glucose, HbA_1c_, and fasting plasma glucose titration target) for study arms in the present analysis. Supplementary Figure S3: linear regression analysis relating baseline and end-of-study fasting plasma glucose to corresponding HbA_1c_ concentrations of studies included in the present analysis. Supplementary Table S5: study duration, titration intervals, and exploratory endpoints at the end of the study for study arms in the present analysis. Supplementary Figure S4: achievement of fasting plasma glucose titration targets in our systematic analysis. Supplementary Table S6: fasting plasma glucose and HbA1c concentrations as well as HbA_1c_ target achievement in clinical trials of basal insulin titration in insulin-naïve patients with type 2 diabetes using various basal insulin preparations in addition to well-defined single or combined oral glucose-lowering agents with different numbers of occasions for titrating basal insulin during the trials included in the present analysis. Supplementary Table S7: insulin dose, hypoglycemia (any or severe), and body weight change from baseline in clinical trials of basal insulin titration in insulin-naïve patients with type 2 diabetes using various basal insulin preparations in addition to well-defined single or combined oral glucose-lowering agents with different numbers of occasions for titrating basal insulin during the trials included in the present analysis. Supplementary Table S8: fasting plasma glucose and HbA_1c_ concentrations as well as HbA_1c_ target achievement in clinical trials of basal insulin titration in insulin-naïve patients with type 2 diabetes using various basal insulin preparations in addition to well-defined single or combined oral glucose-lowering agents, using titration algorithms with varying “stringency” to address hyperglycemia. Supplementary Table S9: results achieved in clinical trials of basal insulin titration in insulin-naïve patients with type 2 diabetes using various basal insulin preparations in addition to well-defined single or combined oral glucose-lowering agents, using different titration algorithms. Supplementary Table S10: heterogeneity of fasting plasma glucose and HbA1c after basal insulin titration with various glucose-lowering medications used in insulin-native patients with type 2 diabetes aiming for different fasting plasma glucose titration targets in studies included in the present analysis.
