# Supplementary material for: Impact of the Fasting Plasma Glucose Titration Target on the Success of Basal Insulin Titration in Insulin-Naïve Patients with Type 2 Diabetes: A Systematic Analysis
Source: J Diabetes Res. 2022 Jul 30;2022:4758042. doi: 10.1155/2022/4758042 (PMC9356801; doi:10.1155/2022/4758042)
Supplement: Supplementary Materials — Supplementary Table S1: detailed presentation of search terms used for the PubMed search to identify studies included in the present systematic analysis investigating the impact of the fasting plasma glucose titration target on the success of basal insulin titration in insulin-naïve patients with type 2 diabetes. Supplementary Figure S1: flow diagram (according to the PRISMA statement) of the search process, selection, and exclusion of publications, which were identified through a systematic PubMed search and included in the present analysis. Data extraction form. Supplementary Table S2: the Jadad score estimating the study quality of the randomized controlled trials included in the present analysis. Supplementary Figure S2: risk of bias as assessed with the “Risk of Bias tool” 2.0 proposed by the Cochrane Collection for all studies included in the present systematic analysis. Supplementary Table S3: patients' baseline characteristics (age, sex, BMI, and body weight) for study arms in the present analysis. Supplementary Table S4: patients' baseline characteristics (duration of diabetes, fasting plasma glucose, HbA1c, and fasting plasma glucose titration target) for study arms in the present analysis. Supplementary Figure S3: linear regression analysis relating baseline and end-of-study fasting plasma glucose to corresponding HbA1c concentrations of studies included in the present analysis. Supplementary Table S5: study duration, titration intervals, and exploratory endpoints at the end of the study for study arms in the present analysis. Supplementary Figure S4: achievement of fasting plasma glucose titration targets in our systematic analysis. Supplementary Table S6: fasting plasma glucose and HbA1c concentrations as well as HbA1c target achievement in clinical trials of basal insulin titration in insulin-naïve patients with type 2 diabetes using various basal insulin preparations in addition to well-defined single or combined oral glucose-lowering agents with diff [file 4758042.f1.docx]

**Online Supplement**

**Impact of the fasting plasma glucose titration target on the success of basal insulin titration in insulin-naïve patients with type 2 diabetes: A systematic analysis**

Jannik Wolters, M.D., Dominik Wollenhaupt, cand. med., Mirna Abd El Aziz, M.D.^1^, Michael A. Nauck, M.D.^1^

^1^ Diabetes Division, Katholisches Klinikum Bochum, St. Josef-Hospital, Ruhr-University Bochum, Bochum, Germany

| ***List of Content*** |
| --- |
| - **Supplementary Table S 1.** Detailed presentation of search terms used for the PubMed search to identify studies included in the present systematic-analysis investigating the impact of the fasting plasma glucose titration target on the success of basal insulin titration in insulin-naïve patients with type 2 diabetes. - **Supplementary Figure S 1.** Flow diagram (according to the Prisma statement) of search process, selection, and exclusion of publications, which were identified through a systematic PubMed search and included in the present analysis. - **Data extraction form.** - **Supplementary Table S 2**. Jadad Score’ estimating the study quality of the randomized controlled trials included in the present analysis. - **Supplementary Figure S 2**. Risk of bias as assessed with the “Risk-of-Bias-Tool” 2.0 proposed by the Cochrane Collection for all studies included in the present systematic analysis. - **Supplementary Table S 3.** Patients´ baseline characteristics (Age, sex, BMI, and body weight) for study arms in the present analysis - **Supplementary Table S 4.** Patients´ baseline characteristics (Duration of diabetes, fasting plasma glucose, HbA1c and fasting plasma glucose titration target) for study arms in the present analysis. - **Supplementary Figure S 3**. Linear regression analysis relating baseline and end-of-study fasting plasma glucose with corresponding HbA1c concentrations of studies included in the present analysis. - **Supplementary Table S 5.** Study duration, titration intervals and exploratory endpoints at study end for study arms in the present analysis. - **Supplementary Figure S 4**. Achievement of fasting plasma glucose titration targets in our systematic analysis. - **Supplementary Table S6.** Fasting plasma glucose and HbA_1c_ concentrations as well as HbA_1c_ target achievement in clinical trials of basal insulin titration in insulin-naïve patients with type 2 diabetes using various basal insulin preparations in addition to well-defined single or combined oral glucose-lowering agents with different numbers of occasions for titrating basal insulin during the trials included in the present analysis. - **Supplementary Table S7.** Insulin dose, hypoglycemia (any or severe) and body weight change from baseline in clinical trials of basal insulin titration in insulin-naïve patients with type 2 diabetes using various basal insulin preparations in addition to well-defined single or combined oral glucose-lowering agents with different numbers of occasions for titrating basal insulin during the trials included in the present analysis. - **Supplementary Table S8.** Fasting plasma glucose and HbA_1c_ concentrations as well as HbA_1c_ target achievement in clinical trials of basal insulin titration in insulin-naïve patients with type 2 diabetes using various basal insulin preparations in addition to well-defined single or combined oral glucose-lowering agents, using titration algorithms with varying “stringency” to address hyperglycemia. - **Supplementary Table S9**. Results achieved in clinical trials of basal insulin titration in insulin-naïve patients with type 2 diabetes using various basal insulin preparations in addition to well-defined single or combined oral glucose-lowering agents, using different titration algorithms. - **Supplementary Table S10.** Heterogeneity of fasting plasma glucose and HbA_1c_ after basal insulin titration with various glucose-lowering medications used in insulin-native Patients with Type 2 diabetes aiming for different fasting plasma glucose titration targets in studies included in the present analysis. |

| **Supplementary Table S 1**. Detailed presentation of search terms used for the PubMed search to identify studies included in the present systematic-analysis investigating the impact of the fasting plasma glucose titration target on the success of basal insulin titration in insulin-naïve patients with type 2 diabetes | |
| --- | --- |
| **Any term from column one associated with any term from columns two in “all fields”** | |
| - Basal insulin - Glargine - Long-acting insulin - Intermediate acting insulin - NPH - Hagedorn - Lantus - Detemir - Levemir - Degludec - Tresiba - Biosimilar insulin - Abasaglar - Toujeo | - Clinical trial - Type 2 diabetes |


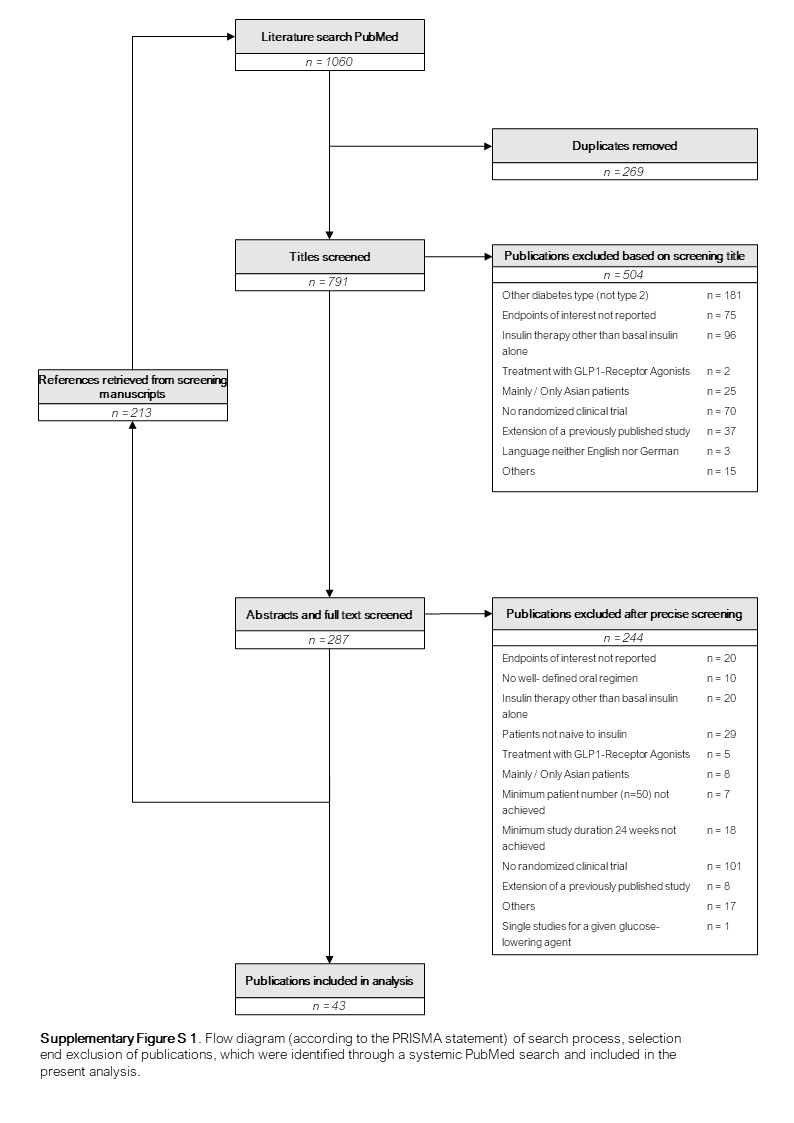


**Data extraction form**. A data extraction form in German language was used for the present analysis.

| **Supplementary Table S 2.** Jadad Score estimating the study quality of the randomized controlled trials included in the present meta-analysis comparing different fasting plasma glucose titration targets in type 2 diabetic patients treated with basal insulin in combination with oral glucose-lowering drugs | | | | | | |
| --- | --- | --- | --- | --- | --- | --- |
| Study /publication | Study described as randomized | Study described as double blind | Withdrawals and dropouts described | Appropriate description of details regarding randomization and double blinding | Either randomization or double blinding inappropriate (- 1 point) | Sum |
| **FPG Titration target ≤ 5.0 mmol/l** | |  |  |  |  |  |
| Gough et al. 2014 (1) | 1 | 0 | 1 | 1 | 0 | 3 |
| Niswender et al. 2014 a (2) | 1 | 0 | 1 | 1 | 0 | 3 |
| Niswender et al. 2014 b (2) | 1 | 0 | 1 | 1 | 0 | 3 |
| Zinman et al. 2013 b (3) | 1 | 0 | 1 | 1 | 0 | 3 |
| Zinman et al. 2013 d (3) | 1 | 0 | 1 | 1 | 0 | 3 |
| Zinman et al. 2012 a (4) | 1 | 0 | 1 | 1 | 0 | 3 |
| Zinman et al. 2012 b (4) | 1 | 0 | 1 | 1 | 0 | 3 |
| Gough et al. 2013 a (5) | 1 | 0 | 1 | 1 | 0 | 3 |
| Gough et al. 2013 b (5) | 1 | 0 | 1 | 1 | 0 | 3 |
| Meneghini et al. 2013 a (6) | 1 | 0 | 1 | 1 | 0 | 3 |
| Meneghini et al. 2013 b (6) | 1 | 0 | 1 | 1 | 0 | 3 |
| Philis-Tsimikas et al. 2019 a (7) | 1 | 0 | 1 | 1 | 0 | 3 |
| Philis-Tsimikas et al. 2013 I (8) | 1 | 0 | 1 | 1 | 0 | 3 |
| Kumar et. al. 2016 (9) | 1 | 0 | 1 | 1 | 0 | 3 |
| Philis-Tsimikas et al. 2013 IIa (10) | 1 | 0 | 1 | 1 | 0 | 3 |
| Philis-Tsimikas et al. 2013 IIb (10) | 1 | 0 | 1 | 1 | 0 | 3 |
| **All studies in this group** | **1.0 ± 0.0** | **0.0 ± 0.0** | **1.0 ± 0.0** | **1.0 ± 0.0** | **0.0 ± 0.0** | **3.0 ± 0.0** |
| **FPG Titration target 5.01-5.6 mmol/l** | |  |  |  |  |  |
| Aroda et al. 2017 (11) | 1 | 0 | 1 | 1 | 0 | 3 |
| Bolli et al. 2015 a (12) | 1 | 0 | 1 | 1 | 0 | 3 |
| Bolli et al. 2015 b (12) | 1 | 0 | 1 | 1 | 0 | 3 |
| D'Alessio et al. 2015 (13) | 1 | 0 | 1 | 1 | 0 | 3 |
| Davies et al. 2013 (14) | 1 | 0 | 1 | 1 | 0 | 3 |
| Diamant et al. 2010 (15) | 1 | 0 | 1 | 1 | 0 | 3 |
| Fritsche et al. 2003 a (16) | 1 | 0 | 1 | 1 | 0 | 3 |
| Fritsche et al. 2003 b (16) | 1 | 0 | 1 | 1 | 0 | 3 |
| Fritsche et al. 2003 c (16) | 1 | 0 | 1 | 1 | 0 | 3 |
| Heine et al. 2005 (17) | 1 | 0 | 1 | 1 | 0 | 3 |
| Janka et al. 2005 (18) | 1 | 0 | 1 | 1 | 0 | 3 |
| Riddle et al. 2013 (19) | 1 | 1 | 1 | 1 | 0 | 4 |
| Weissman et al. 2014 (20) | 1 | 0 | 1 | 1 | 0 | 3 |
| Yki-Järvinen et al. 2006 (21) | 1 | 0 | 1 | 1 | 0 | 3 |
| Aschner et al. 2012 (22) | 1 | 0 | 1 | 1 | 0 | 3 |
| Riddle et al. 2003 a (23) | 1 | 0 | 1 | 1 | 0 | 3 |
| Riddle et al. 2003 b (23) | 1 | 0 | 1 | 1 | 0 | 3 |
| Blicklé et al. 2009 (24) | 1 | 0 | 1 | 1 | 0 | 3 |
| Home et al. 2015 a (25) | 1 | 0 | 1 | 1 | 0 | 3 |
| Home et al. 2015 b (25) | 1 | 0 | 1 | 1 | 0 | 3 |
| Russel-Jones et al. 2009 (26) | 1 | 1 | 1 | 1 | 0 | 4 |
| Giorgino et al. 2015 (27) | 1 | 1 | 1 | 1 | 0 | 4 |
| Davies et al. 2009 (28) | 1 | 0 | 1 | 1 | 0 | 3 |
| Davies et al. 2016 a (29) | 1 | 1 | 1 | 1 | 0 | 4 |
| Davies et al. 2016 b (29) | 1 | 1 | 1 | 1 | 0 | 4 |
| Esposito et al. 2008 a (30) | 1 | 0 | 1 | 1 | 0 | 3 |
| Esposito et al. 2008 b (30) | 1 | 0 | 1 | 1 | 0 | 3 |
| Buse et al. 2009 (31) | 1 | 0 | 1 | 1 | 0 | 3 |
| Standl et al. 2006 a (32) | 1 | 0 | 1 | 1 | 0 | 3 |
| Standl et al. 2006 b (32) | 1 | 0 | 1 | 1 | 0 | 3 |
| Rosenstock et al. 2016 I (33) | 1 | 0 | 1 | 1 | 0 | 3 |
| Strojek et al. 2010 a (34) | 1 | 0 | 1 | 1 | 0 | 3 |
| Strojek et al. 2010 b (34) | 1 | 0 | 1 | 1 | 0 | 3 |
| Roussel et al. 2019 a (35) | 1 | 1 | 1 | 1 | 0 | 4 |
| Roussel et al. 2019 b (35) | 1 | 1 | 1 | 1 | 0 | 4 |
| Vilsbøll et al. 2020 (36) | 1 | 0 | 1 | 1 | 0 | 3 |
| Bretzel et al. 2008 (37) | 1 | 0 | 1 | 1 | 0 | 3 |
| Rosenstock et al. 2016 II (38) | 1 | 0 | 1 | 1 | 0 | 3 |
| **All studies in this group** | **1.0 ± 0.0** | **0.18 ± 0.0** | **1.0 ± 0.0** | **1.0 ± 0.0** | **0 ± 0.0** | **3.18 ± 0.0** |
| **FPG Titration target ≥ 5.61 mmol/l** | |  |  |  |  |  |
| Raskin et al. 2005 (39) | 1 | 0 | 1 | 1 | 0 | 3 |
| Fogelfeld et al. 2010 a (40) | 1 | 0 | 1 | 1 | 0 | 3 |
| Fogelfeld et al. 2010 b (40) | 1 | 0 | 1 | 1 | 0 | 3 |
| Nauck et al. 2013 (41) | 1 | 0 | 1 | 1 | 0 | 3 |
| Rosenstock et al. 2008 a (42) | 1 | 0 | 1 | 1 | 0 | 3 |
| Rosenstock et al. 2008 b (42) | 1 | 0 | 1 | 1 | 0 | 3 |
| Hollander et. al. 2011 (43) | 1 | 0 | 1 | 1 | 0 | 3 |
| **All studies in this group** | **1.0 ± 0.0** | **0.0 ± 0.0** | **1 ± 0.0** | **1.0 ± 0.0** | **0 ± 0.0** | **3.0 ± 0.0** |
| Subgroups´combined values are described as means ± standard error of the mean. | | | | | | |


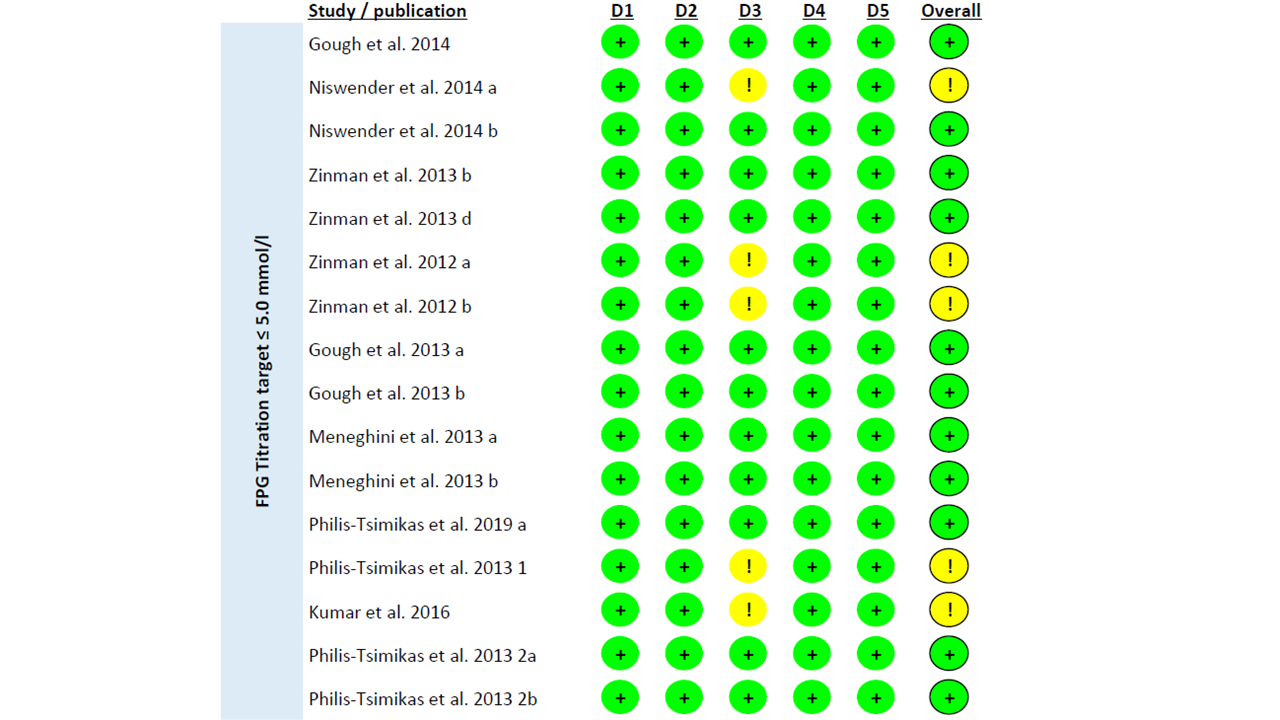

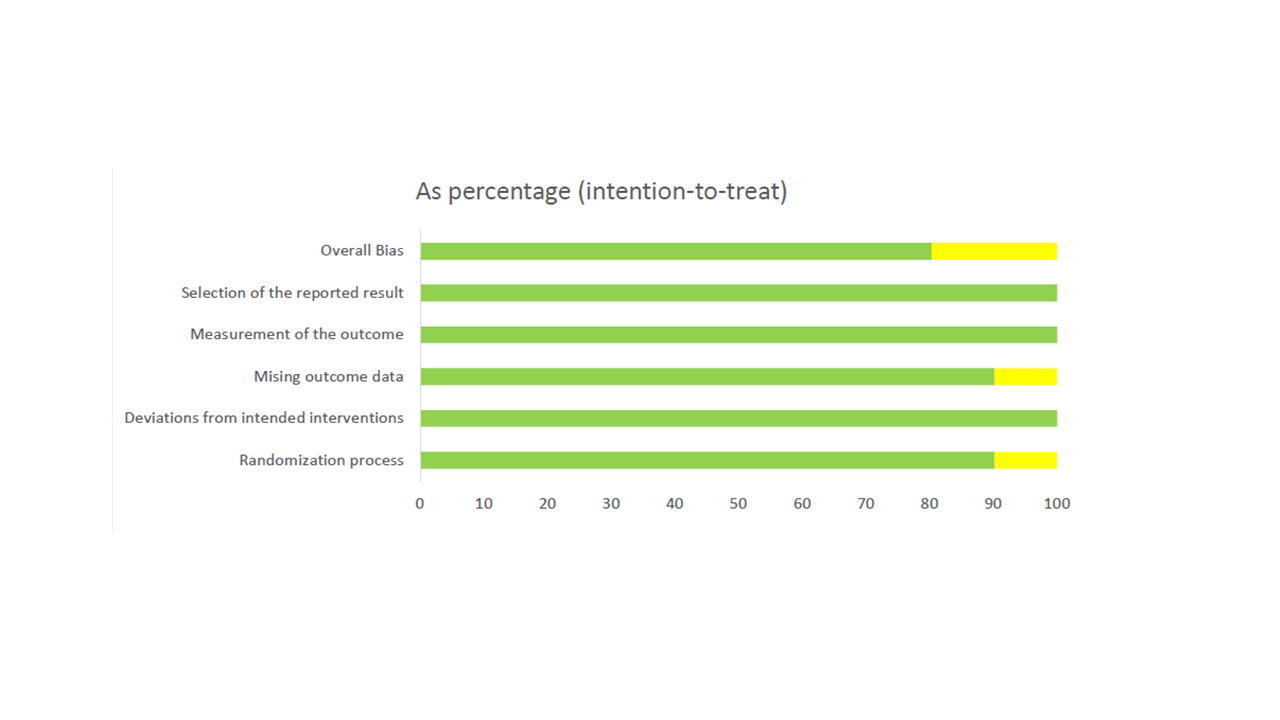

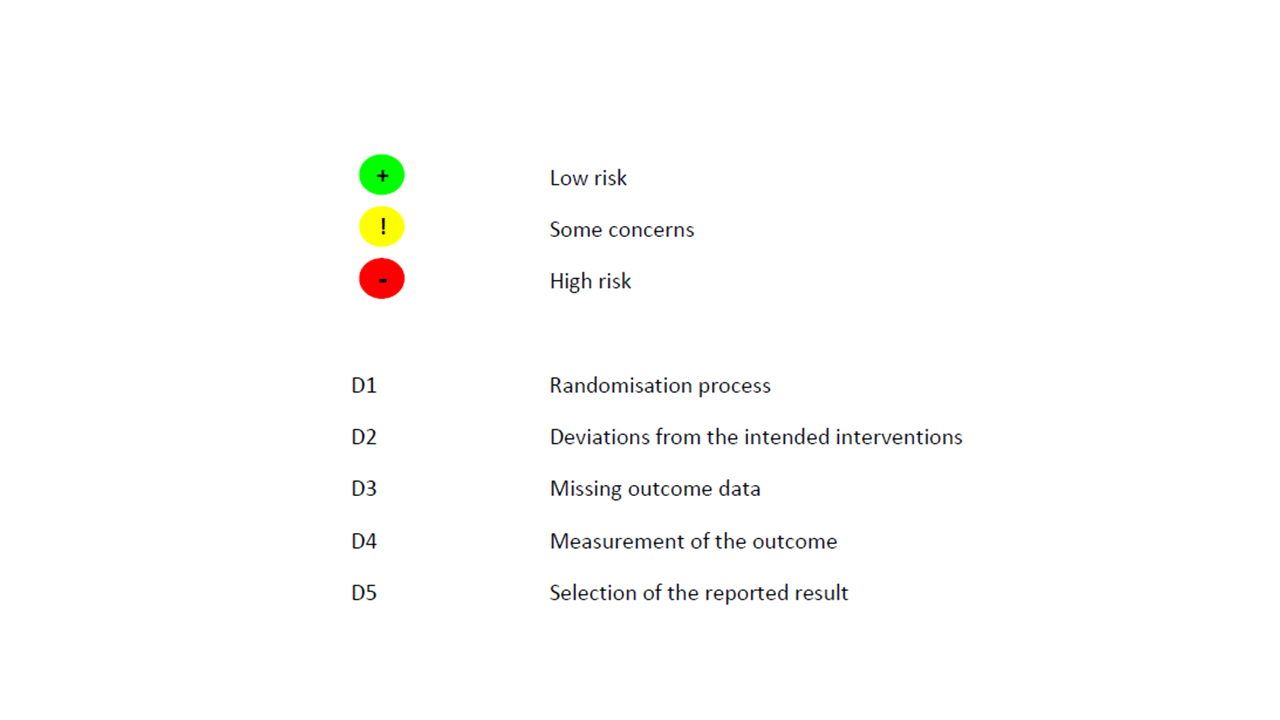

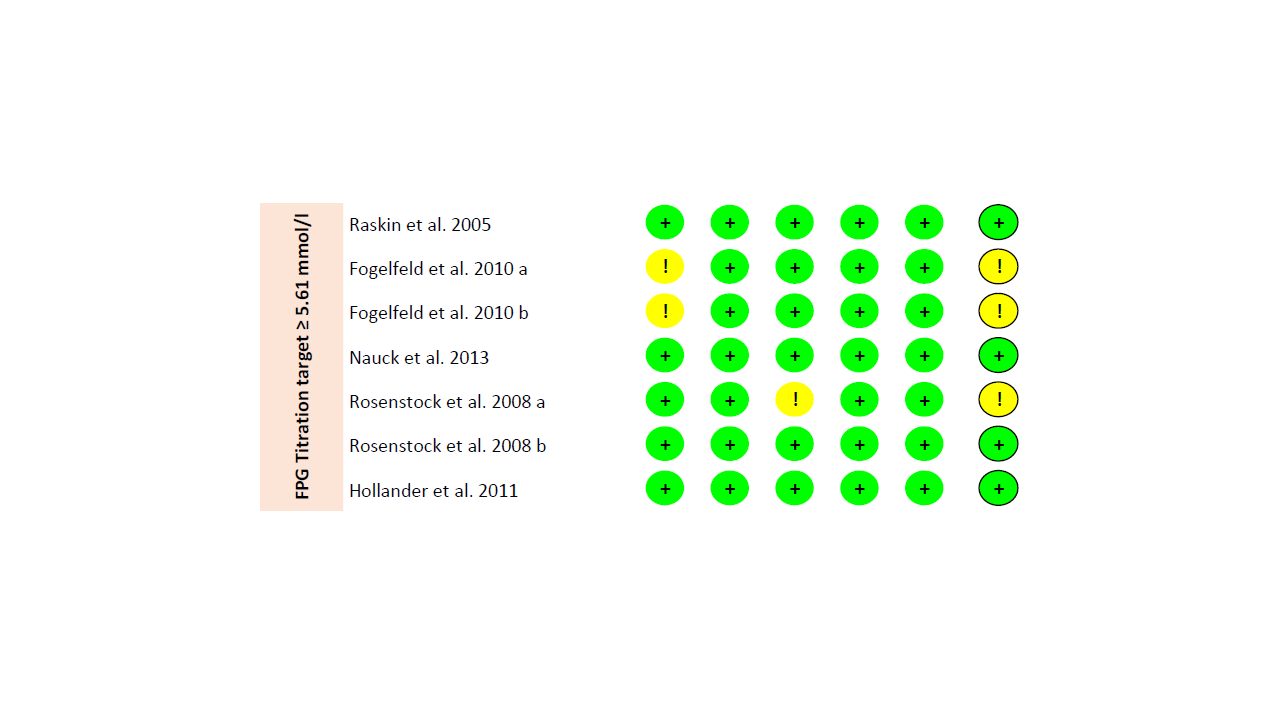

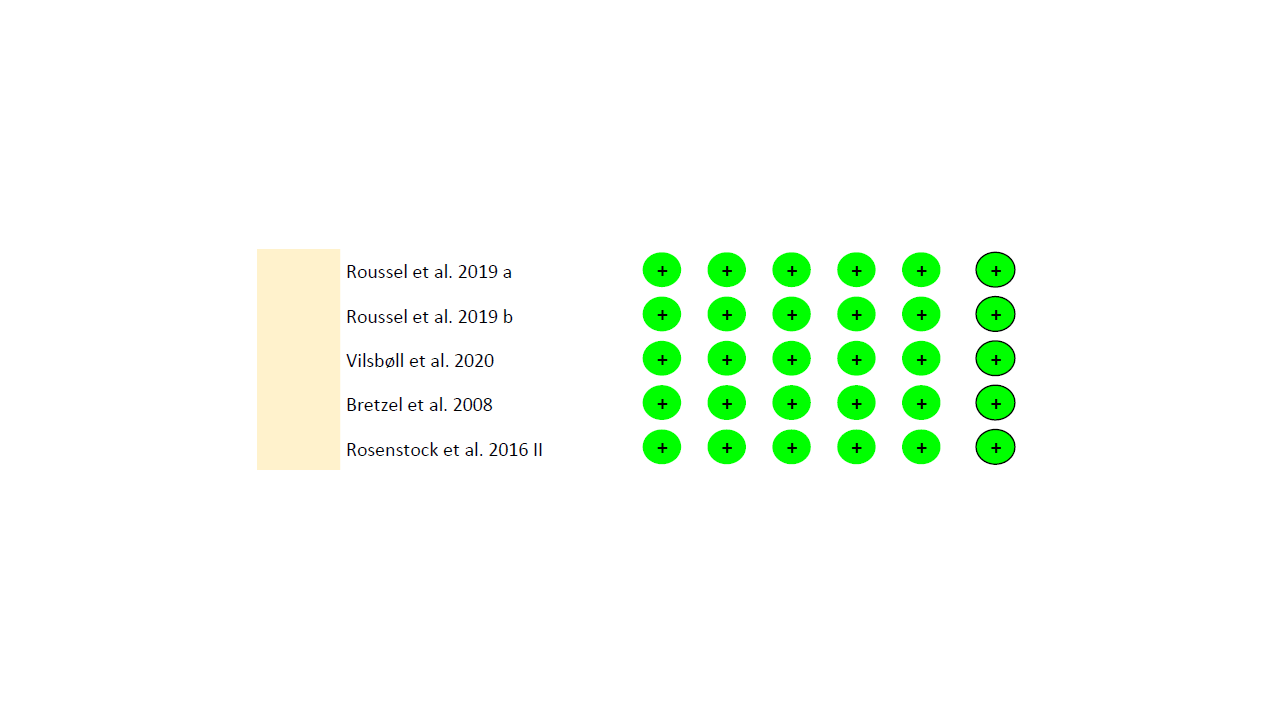

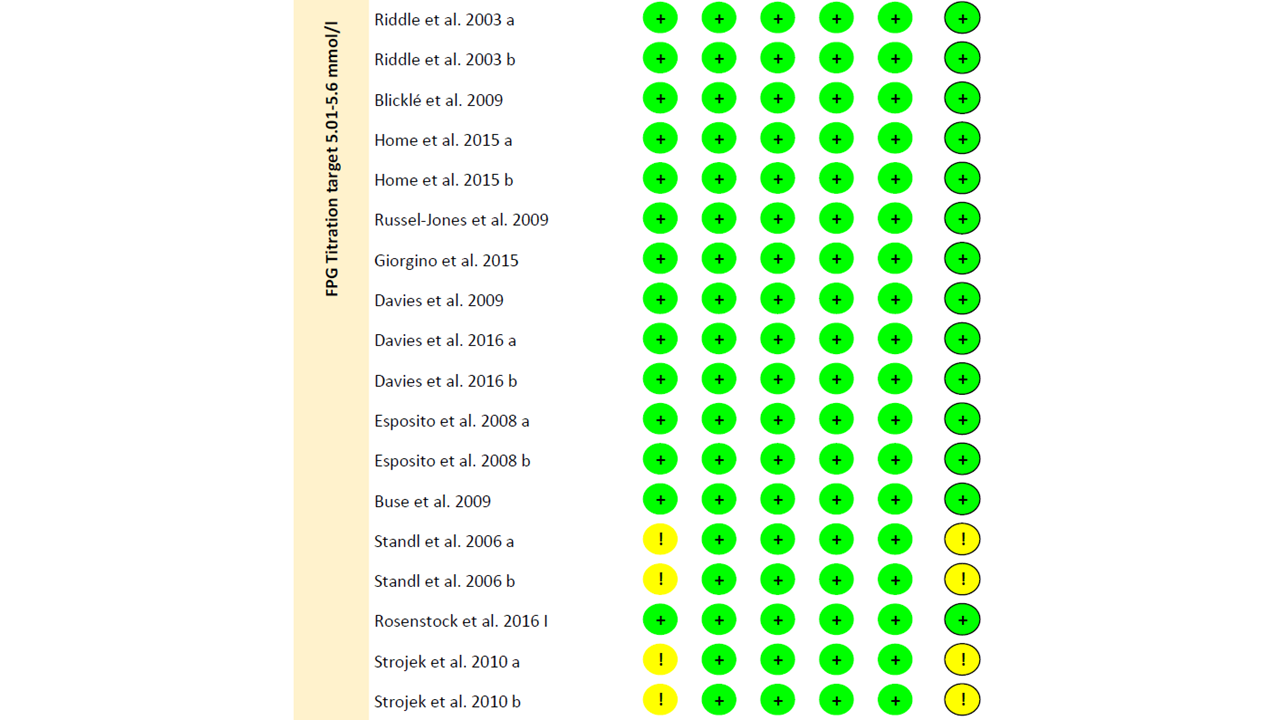

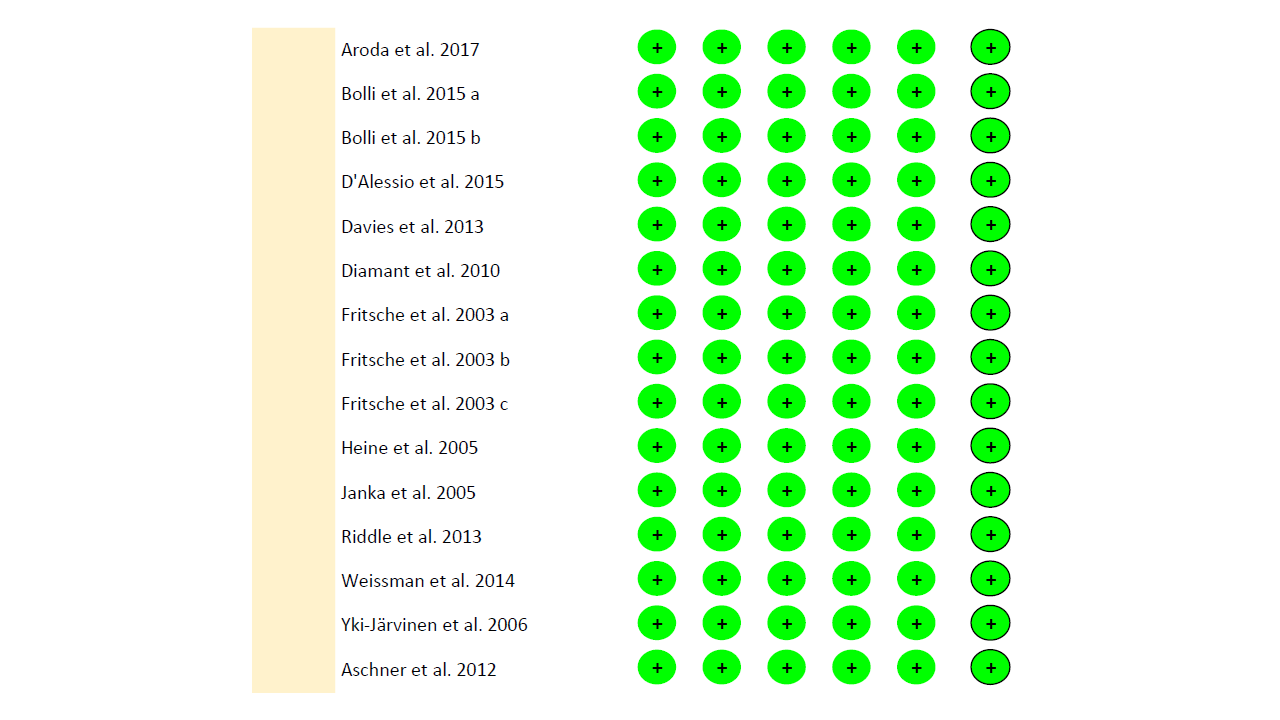

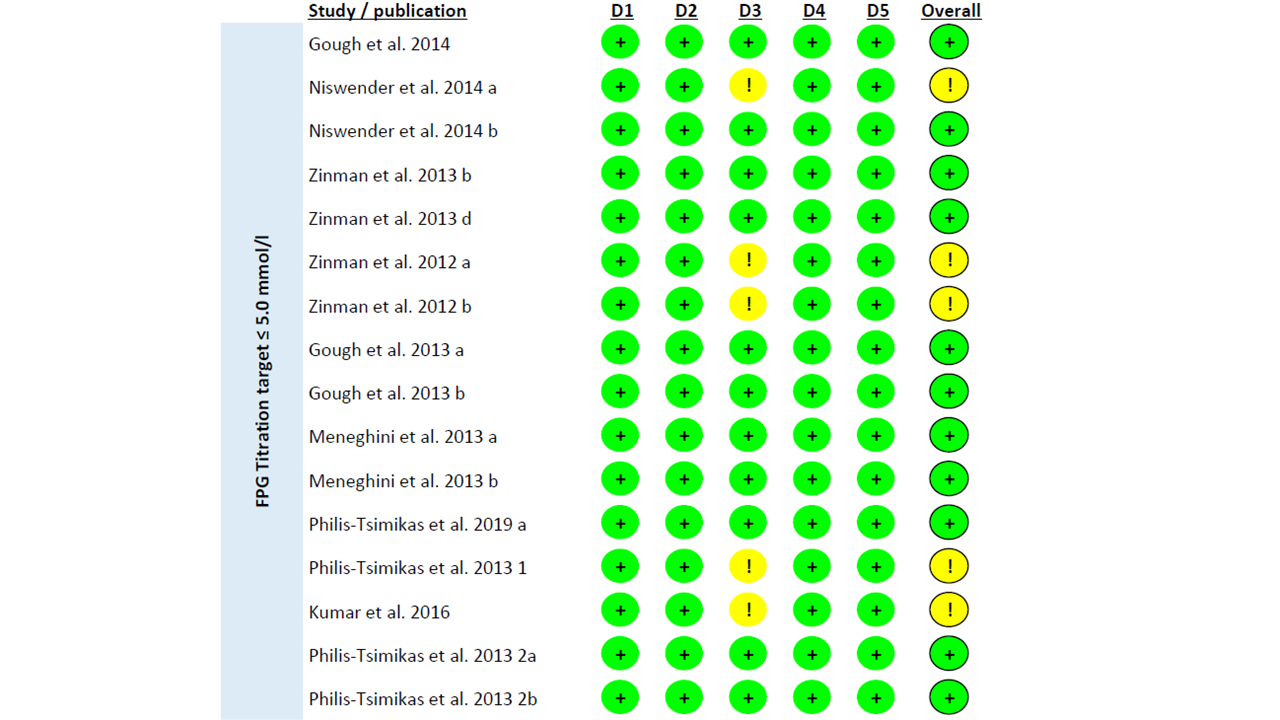


**Supplementary Figure S 2.** Risk of bias as assessed with the “Risk-of-Bias-Tool” 2.0 proposed by the Cochrane Collection for all studies included in the present systematic analysis of different fasting plasma glucose titration targets for titrating basal insulin in insulin-naïve patients with type 2 diabetes.

| **Supplementary Table S 3.** Patients’ baseline characteristics for study arms in the present analysis comparing different fasting plasma glucose titration targets in type 2 diabetic patients treated with basal insulin in combination with oral glucose-lowering drugs | | | | |
| --- | --- | --- | --- | --- |
| Study /publication | Age [years] | Sex  female/male (% female) | BMI [kg/m²] | Body weight [kg] |
| **FPG Titration target ≤ 5.0 mmol/l** |  |  |  |  |
| Gough et al. 2014 (1) | 55 ± 10 | 213/200 (51.6) | 31.2 ± 5.3 | 87.4 ± 19.2 |
| Niswender et al. 2014 a (2) | 57 ± 10 | 143/158 (47.5) | 34.3 ± 5.6 | 97.0 ± 20.4 |
| Niswender et al. 2014 b (2) | 58 ± 10 | 152/153 (49.8) | 34.4 ± 5.4 | 96.4 ± 18.2 |
| Zinman et al. 2013 b (3) | 58 ± 10 | 93/137 (40.4) | 33.0 ± 5.4 | 95.7 ± 19.0 |
| Zinman et al. 2013 d (3) | 58 ± 11 | 99/135 (42.3) | 31.9 ± 5.5 | 91.4 ± 18.7 |
| Zinman et al. 2012 a (4) | 59 ± 10 | 302/464 (39.1) | 30.9 ± 4.8 | 89.4 ± 17.7 |
| Zinman et al. 2012 b (4) | 59 ± 10 | 90/167 (35.0) | 31.6 ± 4.4 | 91.8 ± 15.8 |
| Gough et al. 2013 a (5) | 58 ± 9 | 109/119 (47.8) | 32.2 ± 5.4 | 92.2 ± 18.5 |
| Gough et al. 2013 b (5) | 57 ± 9 | 105/124 (45.9) | 32.7 ± 5.3 | 92.7 ± 18.4 |
| Meneghini et al. 2013 a (6) | 57 ± 10 | 97/129 (43.0) | 28.9 ± 4.0 | 82.8 ± 17.2 |
| Meneghini et al. 2013 b (6) | 57 ± 10 | 100/127 (44.0) | 29.1 ± 3.9 | 81.7 ± 16.2 |
| Philis-Tsimikas et al. 2019 a (7) | 57 ± 10 | 84/126 (40.0) | 30.9 ± 4.8 | 87.2 ± 17.2 |
| Philis-Tsimikas et al. 2013 I (8) | 56 ± 10 | 84/141 (37.3) | 30.0 ± 5.1 | 83.9 ± 19.3 |
| Kumar et. al. 2016 (9) | 56 ± 9 | 127/136 (48.3) | 30.5 ± 5.1 | 85.1 ± 18.6 |
| Philis-Tsimikas et al. 2013 IIa (10) | 59± 10 | 43/68 (38.7) | 33.4 ± 5.1 | 95.7 ± 18.9 |
| Philis-Tsimikas et al. 2013 IIb (10) | 59 ± 11 | 36/75 (32.4) | 31.5 ± 5.2 | 91.3 ± 18.2 |
| **All studies in this group** | **58 ± 10** | **1877/2459 (43.3)** | **31.6 ± 5.1** | **90.1 ± 18.2** |
| **FPG Titration target 5.01-5.6 mmol/l** | |  |  |  |
| Aroda et al. 2017 (11) | 56 ± 11 | 165/195 (45.8) | 33.0 ± 6.5 | 92.6 ± 21.5 |
| Bolli et al. 2015 a (12) | 58 ± 10 | 186/253 (42.4) | 32.8 ± 6.9 | 95.1 ± 23.3 |
| Bolli et al. 2015 b (12) | 57 ± 10 | 185/254 (42.1) | 33.2 ± 6.6 | 95.6 ± 22.6 |
| D'Alessio et al. 2015 (13) | 57 ± 9 | 224/250 (47.3) | 32.0 ± 4.2 | 90.8 ± 16.6 |
| Davies et al. 2013 (14) | 58 ± 10 | 33/72 (31.4) | 33.7 ± 4.7 | 97.9 ± 15.8 |
| Diamant et al. 2010 (15) | 58 ± 9 | 100/123 (45.0) | 32.0 ± 5.0 | 90.6 ± 16.4 |
| Fritsche et al. 2003 a (16) | 62 ± 9 | 113/119 (48.7) | 28.9 ± 3.9 | 81.0 ± 14.9 |
| Fritsche et al. 2003 b (16) | 60 ± 9 | 95/132 (41.9) | 28.7 ± 3.9 | 82.1 ± 13.6 |
| Fritsche et al. 2003 c (16) | 61 ± 9 | 114/122 (48.3) | 28.6 ± 4.5 | 80.7 ± 15.8 |
| Heine et al. 2005 (17) | 58 ± 10 | 116/151 (43.4) | 31.3 ± 4.6 | 88.3 ± 17.9 |
| Janka et al. 2005 (18) | 61 ± 9 | 69/108 (39.0) | 29.5 ± 3.6 | 85.1 ± 14.7 |
| Riddle et al. 2013 (19) | 56± 10 | 110/113 (49.3) | 32.0 ± 6.6 | 87.3 ± 21.8 |
| Weissman et al. 2014 (20) | 55 ± 10 | 109/132 (45.2) | 33.0 ± 5.4 | 94.6 ± 19.1 |
| Yki-Järvinen et al. 2006 (21) | 56 ± 8 | 23/38 (38.0) | 31.3 ± 5.5 | 92.0 ± 18.7 |
| Aschner et al. 2012 (22) | 54 ± 9 | 113/114 (50.0) | 31.1 ± 4.9 | 83.4 ± 18.2 |
| Riddle et al. 2003 a (23) | 55 ± 10 | 165/202 (45.0) | 32.5 ± 4.6 | n.r. |
| Riddle et al. 2003 b (23) | 56 ± 9 | 171/218 (44.0) | 32.2 ± 4.8 | n.r. |
| Blicklé et al. 2009 (24) | 61 ± 8 | 46/57 (45.0) | 30.1 ± 3.5 | 85.0 ± 12.5 |
| Home et al. 2015 a (25) | 57 ± 8 | 198/154 (56.2) | 29.7 ± 4.5 | 81.2 ± 16.0 |
| Home et al. 2015 b (25) | 57 ± 8 | 195/154 (55.9) | 30.1 ± 4.5 | 82.7 ± 15.5 |
| Russel-Jones et al. 2009 (26) | 58 ± 11 | 93/139 (40.0) | 30.3 ± 5.3 | 85.0 ± 17.9 |
| Giorgino et al. 2015 (27) | 57 ± 9 | 128/134 (49.0) | 32.0 ± 6.0 | 88.0 ± 20.0 |
| Davies et al. 2009 (28) | 56 ± 8 | 39/77 (33.6) | 33.7 ± 4.9 | 97.6 ± 16.4 |
| Davies et al. 2016 a (29) | 59 ± 10 | 227/308 (42.4) | 32.0 ± 5.2 | 90.9 ± 18.7 |
| Davies et al. 2016 b (29) | 59 ± 10 | 453/550 (45.2) | 32.2 ± 5.2 | 91.0 ± 18.4 |
| Esposito et al. 2008 a (30) | 54 ± 7 | 27/28 (49.1) | 29.7 ± 4.3 | 84.8 ± 13.0 |
| Esposito et al. 2008 b (30) | 55 ± 7 | 26/29 (47.3) | 29.4 ± 4.6 | 94.1 ± 13.0 |
| Buse et al. 2009 (31) | 57± 10 | 494/552 (47.2) | 32.0 ± 6.0 | 88.0 ± 21.0 |
| Standl et al. 2006 a (32) | 62 ± 10 | 142/170 (45.5) | 28.2 ± 4.0 | n.r. |
| Standl et al. 2006 b (32) | 62 ± 10 | 142/170 (45.5) | 28.7 ± 3.9 | n.r. |
| Rosenstock et al. 2016 I (33) | 58 ± 9 | 230/237 (49.3) | 31.7 ± 4.5 | 89.8 ± 16.3 |
| Strojek et al. 2010 a (34) | 58 ± 9 | 107/122 (46.7) | 30.7 ± 5.0 | 84.2 ± 16.8 |
| Strojek et al. 2010 b (34) | 57 ± 9 | 116/113 (50.7) | 31.6 ± 5.5 | 86.1 ± 18.6 |
| Roussel et al. 2019 a (35) | 59 ± 10 | 203/170 (54.4) | 31.2 ± 5.8 | 84.8 ± 19.8 |
| Roussel et al. 2019 b (35) | 58 ± 10 | 180/190 (48.6) | 31.1 ± 5.7 | 85.6 ± 18.9 |
| Vilsbøll et al. 2020 (36) | 55 ± 10 | 148/171 (46.4) | 32.0 ± 5.4 | 89.4 ± 18.4 |
| Bretzel et al. 2008 (37) | 60 ± 9 | 84/102 (45.0) | 29.2 ± 3.6 | 84.1 ± 15.0 |
| Rosenstock et al. 2016 II (38) | 57 ± 9 | 77/85 (47.5) | 32.0 ± 4.4 | 91.6 ± 16.7 |
| **All studies in this group** | **58 ± 9** | **5446/6308 (46.3)** | **31.4 ± 5.2** | **88.6 ± 18.5** |
| **FPG Titration target ≥ 5.61 mmol/l** |  |  |  |  |
| Raskin et al. 2005 (39) | 52 ± 10 | 51/65 (44.0) | 31.4 ± 5.3 | 89.9 ± 19.0 |
| Fogelfeld et al. 2010 a (40) | 56 ± 10 | 108/111 (49.3) | 30.0 ± 5.0 | 81.1 ± 17.5 |
| Fogelfeld et al. 2010 b (40) | 56 ± 10 | 97/113 (46.2) | 30.1 ± 5.1 | 82.7 ± 19.3 |
| Nauck et al. 2013 (41) | 58 ± 9 | 171/148 (53.6) | 32.7 ± 5.2 | 90.6 ± 19.5 |
| Rosenstock et al. 2008 a (42) | 58 ± 10 | 125/166 (43.0) | 30.6 ± 4.8 | 87.4 ± 16.6 |
| Rosenstock et al. 2008 b (42) | 59 ± 10 | 120/171 (41.2) | 30.5 ± 4.6 | 87.4 ± 17.4 |
| Hollander et. al. 2011 (43) | 57 ± 10 | 39/68 (36.0) | 31.8 ± 5.2 | 93.1 ± 20.2 |
| **All studies in this group** | **58 ± 10** | **711/842 (45.8)** | **31.0 ± 5.0** | **88.6 ± 18.2** |
| **All studies** | **58 ± 10** | **8034/9609 (45.5)** | **31.4 ± 5.2** | **88.8 ± 18.4** |
| Continuous variables presented as means ± standard deviation, categorical variables are presented as number fulfilling/not  fulfilling the criterion and the proportion (percentage) fulfilling the criterion in question. Combined means and standard deviations for all studies belonging to one titration target category were calculated with standard equations. FPG: Fasting plasma glucose; n.r.: not reported. | | | | |

| **Supplementary Table S 4.** Patients’ baseline characteristics for study arms in the present analysis comparing different fasting plasma glucose titration targets in type 2 diabetic patients treated with basal insulin in combination with oral glucose-lowering drugs | | | | | |
| --- | --- | --- | --- | --- | --- |
| Study /publication | Duration of Diabetes [y] | HbA_1c_ [%] | HbA_1c_ [mmol/mol] | FPG [mmol/l] | Titration target as defined for a given study [mmol/l] |
| **FPG Titration target ≤ 5.0 mmol/l** |  |  |  |  |  |
| Gough et al. 2014 (1) | 7 ± 5 | 8.3 ± 1.0 | 67.3 ± 10.7 | 9.4 ± 2.7 | 5.0 |
| Niswender et al. 2014 a (2) | 9 ± 6 | 7.9 ± 0.6 | 63.2 ± 6.8 | 9.2 ± 2.0 | 5.0 |
| Niswender et al. 2014 b (2) | 9 ± 6 | 8.0 ± 0.7 | 63.5 ± 7.3 | 9.3 ± 2.3 | 5.0 |
| Zinman et al. 2013 b (3) | 9 ± 6 | 8.3 ± 0.9 | 67.2 ± 9.8 | 9.6 ± 2.4 | 5.0 |
| Zinman et al. 2013 d (3) | 9 ± 1 | 8.3 ± 0.8 | 67.2 ± 8.7 | 9.9 ± 2.4 | 5.0 |
| Zinman et al. 2012 a (4) | 9 ± 6 | 8.2 ± 0.8 | 66.1 ± 8.7 | 9.6 ± 2.6 | 4.9 |
| Zinman et al. 2012 b (4) | 9 ± 6 | 8.2 ± 0.8 | 66.1 ± 8.7 | 9.7 ± 2.6 | 4.9 |
| Gough et al. 2013 a (5) | 8 ± 7 | 8.3 ± 1.0 | 67.1 ± 10.7 | 9.6 ± 2.9 | 4.9 |
| Gough et al. 2013 b (5) | 8 ± 6 | 8.2 ± 0.9 | 66.6 ± 9.4 | 9.7 ± 2.6 | 4.9 |
| Meneghini et al. 2013 a (6) | 8 ± 6 | 8.0 ± 0.6 | 63.5 ± 6.8 | 8.7 ± 2.3 | 5.0 |
| Meneghini et al. 2013 b (6) | 8 ± 7 | 7.9 ± 0.6 | 62.4 ± 6.3 | 8.5 ± 2.2 | 5.0 |
| Philis-Tsimikas et al. 2019 a (7) | 9 ± 6 | 8.4 ± 1.1 | 68.3 ± 12.0 | 9.6 ± 2.4 | 5.0 |
| Philis-Tsimikas et al. 2013 I (8) | 8 ± 6 | 8.8 ± 1.0 | 72.7 ± 10.9 | 9.4 ± 2.6 | 5.0 |
| Kumar et. al. 2016 (9) | 10 ± 6 | 8.9 ± 0.9 | 73.8 ± 9.8 | 10.4 ± 2.8 | 4.9 |
| Philis-Tsimikas et al. 2013 IIa (10) | 9 ± 6 | 8.1 ± 0.9 | 65.0 ± 9.8 | 9.3 ± 2.6 | 5.0 |
| Philis-Tsimikas et al. 2013 IIb (10) | 10 ± 7 | 8.2 ± 0.9 | 66.1 ± 9.8 | 9.4 ± 2.8 | 5.0 |
| **All studies in this group** | **9 ± 6** | **8.2 ± 0.8** | **66.6 ± 9.3** | **9.5 ± 2.5** | **4.96** |
| **FPG Titration target 5.01-5.6 mmol/l** | |  |  |  |  |
| Aroda et al. 2017 (11) | 9 ± 6 | 8.1 ± 0.9 | 65.4 ± 9.6 | 9.7 ± 2.8 | 5.5 |
| Bolli et al. 2015 a (12) | 10 ± 7 | 8.5 ± 1.0 | 69.5 ± 11.4 | 9.9 ± 2.9 | 5.6 |
| Bolli et al. 2015 b (12) | 10 ± 6 | 8.6 ± 1.1 | 70.1 ±11.7 | 10.2 ± 2.9 | 5.6 |
| D'Alessio et al. 2015 (13) | 9 ± 25 | 9.0 ± 1.0 | 75.0 ± 11.0 | 9.8 ± 2.6 | 5.5 |
| Davies et al. 2013 (14) | 7 ± 5 | 8.4 ± 0.9 | 67.8 ± 9.6 | 9.8 ± 2.3 | 5.5 |
| Diamant et al. 2010 (15) | 8 ± 6 | 8.3 ± 1.0 | 67.2 ± 10.9 | 9.7 ± 2.7 | 5.5 |
| Fritsche et al. 2003 a (16) | 9 ± 10 | 9.1 ± 1.1 | 76.0 ± 12.0 | 12.2 ± 3.2 | 5.6 |
| Fritsche et al. 2003 b (16) | 8 ± 13 | 9.1 ± 1.1 | 76.0 ± 12.0 | 12.0 ± 2.9 | 5.6 |
| Fritsche et al. 2003 c (16) | 9 ± 9 | 9.1 ± 1.1 | 76.0 ± 12.0 | 12.1 ± 3.0 | 5.6 |
| Heine et al. 2005 (17) | 9 ± 6 | 8.3 ± 1.0 | 67.2 ± 10.9 | 10.4 ± 2.9 | 5.6 |
| Janka et al. 2005 (18) | 10 ± 7 | 8.9 ± 1.0 | 73.2 ± 10.7 | 9.5 ± 1.9 | 5.6 |
| Riddle et al. 2013 (19) | 10 ± 6 | 7.6 ± 0.5 | 59.6 ± 5.5 | 10.7± 2.0 | 5.6 |
| Weissman et al. 2014 (20) | 8 ± 5 | 8.4 ± 1.0 | 67.9 ± 10.4 | 9.7 ± 2.9 | 5.6 |
| Yki-Järvinen et al. 2006 (21) | 9 ± 8 | 9.5 ± 0.8 | 80.3 ± 8.5 | 13.0 ± 2.3 | 5.5 |
| Aschner et al. 2012 (22) | 3 ± 3 | 8.5 ± 1.0 | 69.4 ±10.9 | 9.1 ± 2.2 | 5.5 |
| Riddle et al. 2003 a (23) | 8 ± 6 | 8.6 ± 0.9 | 70.6 ± 9.8 | 11.0 ± 2.7 | 5.6 |
| Riddle et al. 2003 b (23) | 9 ± 6 | 8.6 ± 0.9 | 70.1 ± 9.8 | 10.8 ± 2.6 | 5.6 |
| Blicklé et al. 2009 (24) | 10 ± 6 | 7.6 ± 0.3 | 59.6 ± 3.3 | 9.4 ± 1.9 | 5.5 |
| Home et al. 2015 a (25) | 9 ± 6 | 8.2 ± 0.8 | 66.0 ± 9.0 | 9.2 ± 2.1 | 5.5 |
| Home et al. 2015 b (25) | 9 ± 6 | 8.2 ± 0.9 | 66.0 ±10.0 | 9.0 ± 2.0 | 5.5 |
| Russel-Jones et al. 2009 (26) | 9 ± 6 | 8.2 ± 0.9 | 66.1 ± 9.8 | 9.1 ± 2.0 | 5.5 |
| Giorgino et al. 2015 (27) | 9 ± 6 | 8.1 ± 1.0 | 65.0 ± 10.4 | 9.0 ± 2.7 | 5.5 |
| Davies et al. 2009 (28) | 8 ± 4 | 8.5 ± 0.7 | 69.2 ± 7.2 | 10.1 ± 2.2 | 5.6 |
| Davies et al. 2016 a (29) | 11 ± 7 | 8.5 ± 0.8 | 69.0 ± 8.7 | 9.4 ± 2.6 | 5.6 |
| Davies et al. 2016 b (29) | 11 ± 6 | 8.5 ± 0.8 | 69.0 ± 8.7 | 9.4 ± 3.6 | 5.6 |
| Esposito et al. 2008 a (30) | 5 ± 3 | 8.8 ± 0.7 | 72.7 ±7.7 | 10.6 ± 2.3 | 5.6 |
| Esposito et al. 2008 b (30) | 5 ± 3 | 8.7 ± 0.7 | 71.6 ±7.7 | 10.7 ± 2.2 | 5.6 |
| Buse et al. 2009 (31) | 9 ± 6 | 9.0 ± 1.2 | 74.9 ± 13.1 | 10.9 ± 3.1 | 5.5 |
| Standl et al. 2006 a (32) | 10 ± 6 | 8.8 ± 1.0 | 72.9 ± 11.0 | 11.0 ± 3.2 | 5.5 |
| Standl et al. 2006 b (32) | 10 ± 7 | 8.8 ± 1.0 | 72.8 ±10.7 | 11.0 ± 3.4 | 5.5 |
| Rosenstock et al. 2016 I (33) | 9 ± 6 | 8.2 ± 0.7 | 66.0 ± 7.7 | 9.8 ± 2.3 | 5.6 |
| Strojek et al. 2010 a (34) | 10 ± 7 | 8.7 ± 0.7 | 71.3 ± 7.5 | 6.4 ± 1.4 | 5.6 |
| Strojek et al. 2010 b (34) | 10 ± 7 | 8.7 ± 0.7 | 71.3 ± 7.4 | 6.3 ± 1.4 | 5.6 |
| Roussel et al. 2019 a (35) | 10 ± 7 | 8.8 ± 0.9 | 72.5 ± 9.8 | 11.0 ± 2.8 | 5.6 |
| Roussel et al. 2019 b (35) | 11 ± 7 | 8.8 ± 1.0 | 72.7 ± 10.6 | 11.2 ± 2.9 | 5.6 |
| Vilsbøll et al. 2020 (36) | 9 ± 6 | 9.1 ± 1.1 | 76.0 ±12.0 | 10.5 ± 3.0 | 5.6 |
| Bretzel et al. 2008 (37) | 9 ± 7 | 8.7 ± 1.0 | 71.9 ± 10.6 | 10.4 ± 2.0 | 5.5 |
| Rosenstock et al. 2016 II (38) | 7 ± 5 | 8.0 ± 0.8 | 63.9 ± 8.7 | 9.5 ± 2.2 | 5.6 |
| **All studies in this group** | **9 ± 8** | **8.6 ± 0.9** | **70.3 ± 10.3** | **10.2 ± 2.8** | **5.56** |
| **FPG Titration target ≥ 5.61 mmol/l** |  |  |  |  |  |
| Raskin et al. 2005 (39) | 9 ± 5 | 9.8 ± 1.4 | 83.6 ± 15.3 | 13.5 ± 3.8 | 6.1 |
| Fogelfeld et al. 2010 a (40) | 10 ± 6 | 8.8 ± 0.7 | 72.7 ± 7.8 | 7.0 ± 1.8 | 5.7 |
| Fogelfeld et al. 2010 b (40) | 9 ± 6 | 8.8 ± 0.7 | 72.7 ± 7.8 | 6.9 ± 1.8 | 6.2 |
| Nauck et al. 2013 (41) | 10 ± 6 | 8.4 ± 0.9 | 68.0 ± 10.0 | 11.2 ± 2.8 | 6.1 |
| Rosenstock et al. 2008 a (42) | 9 ± 6 | 8.6 ± 0.8 | 70.9 ± 8.5 | 10.8 ± 5.4 | 6.0 |
| Rosenstock et al. 2008 b (42) | 9 ± 6 | 8.6 ± 0.8 | 70.7 ± 8.4 | 10.8 ± 5.5 | 6.0 |
| Hollander et. al. 2011 (43) | 10 ± 6 | 8.5 ± 0.7 | 69.4 ± 7.7 | 9.7 ± 2.2 | 6.0 |
| **All studies in this group** | **9 ± 6** | **8.7 ± 0.8** | **71.7 ± 9.2** | **11.1 ± 4.4** | **6.01** |
| **All studies** | **9 ± 7** | **8.5 ± 0.9** | **69.6 ± 10.0** | **10.1 ± 2.9** |  |
| Continuous variables presented as means ± standard deviation, categorical variables are presented as number fulfilling/not  fulfilling the criterion and the proportion (percentage) fulfilling the criterion in question. Combined means and standard deviations for all studies belonging to one titration target category were calculated with standard equations. FPG: Fasting plasma glucose. | | | | | |


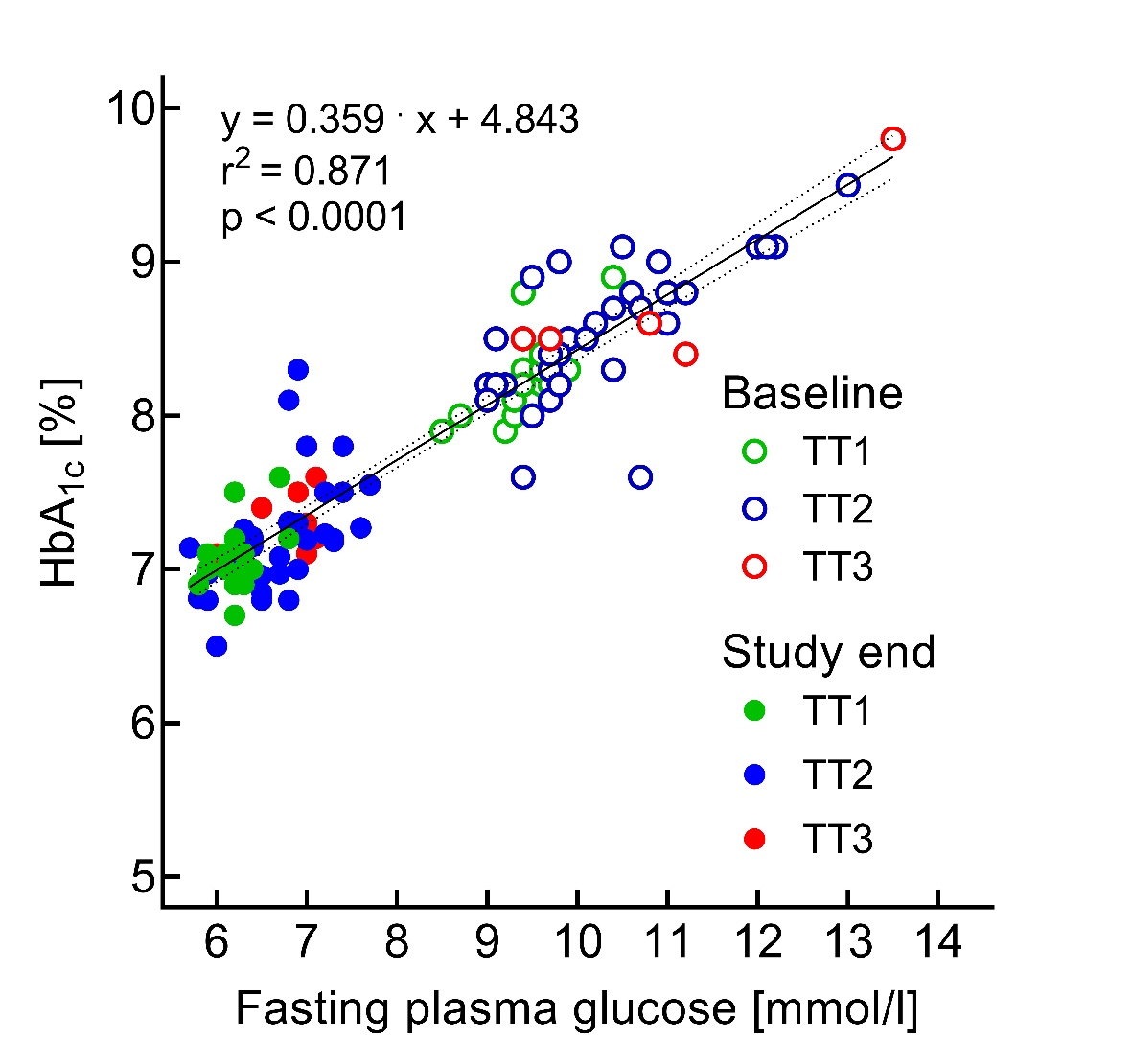


**Supplementary Figure S 3.** Linear regression analysis relating baseline and end-of-study fasting plasma glucose with corresponding HbA_1c_ concentrations. Each symbol represents the mean values for each of the study arms analysed in our systematic analysis of the influence of three categories of fasting plasma glucose titration targets on the success of basal insulin therapy introduced in insulin-naïve patients with type 2 diabetes treated with well-defined single or combined oral glucose-lowering agents.

| **Supplementary Table S 5.** Study duration, titration intervals and exploratory endpoints at study end for study arms in the present analysis comparing different fasting plasma glucose titration targets in type 2 diabetic patients treated with basal insulin in combination with oral glucose-lowering agents. | | | | | |
| --- | --- | --- | --- | --- | --- |
| Study /publication | Study duration [weeks] | Titration interval as defined for a given study | Body weight, change from baseline [kg] | Insulin dose [U/d] | Insulin dose [U/kg/d] |
| **FPG Titration target ≤ 5.0 mmol/l** |  |  |  |  |  |
| Gough et al. 2014 (1) | 26 | Twice a week/ every 3 days | 1.60 ± 4.0 | 52 ± 28 | 0.59 ± 0.31 |
| Niswender et al. 2014 a (2) | 26 | Twice a week/ every 3 days | -0.56 ± 4.0 | 94 ± n.r. | 0.97 ± 0.57 |
| Niswender et al. 2014 b (2) | 26 | Twice a week/ every 3 days | -1.05 ± 4.0 | 91 ± n.r. | 0.95 ± 0.56 |
| Zinman et al. 2013 b (3) | 26 | Weekly | 1.20 ± 3.4* | 62 ± 33 | 0.60 ± 0.30 |
| Zinman et al. 2013 d (3) | 26 | Weekly | 0.50 ± 1.4 | 56 ± 31 | 0.60 ± 0.30 |
| Zinman et al. 2012 a (4) | 52 | During official study visits | 2.40 ± 4.3 | 54 ± n.r. | 0.59 ± 0.35 |
| Zinman et al. 2012 b (4) | 52 | During official study visits | 2.10 ± 4.1 | 56 ± n.r. | 0.60 ± 0.32 |
| Gough et al. 2013 a (5) | 26 | Weekly | 1.90 ± 5.4* | 57 ± n.r. | 0.53 ± 0.31 |
| Gough et al. 2013 b (5) | 26 | Weekly | 1.50 ± 4.3* | 61 ± n.r. | 0.60 ± 0.35 |
| Meneghini et al. 2013 a (6) | 26 | Weekly | -0.49 ± 3.3 | 57 ± 30 | 0.70 ± 0.34 |
| Meneghini et al. 2013 b (6) | 26 | Weekly | 1.00 ± 3.1 | 51 ± 22 | 0.61 ± 0.28 |
| Philis-Tsimikas et al. 2019 a (7) | 26 | Twice a week/ every 3 days | 2.00 ± 3.8 | 54 ± 26 | 0.60 ± 0.29 |
| Philis-Tsimikas et al. 2013 I (8) | 26 | Weekly | 2.28 ± 6.5* | 43 ± n.r. | 0.50 ± 0.29 |
| Kumar et. al. 2016 (9) | 26 | Twice a week/ every 3 days | 2.80 ± 8.0* | 62 ± n.r. | 0.70 ± 0.41 |
| Philis-Tsimikas et al. 2013 IIa (10) | 26 | Weekly | 1.60 ± 4.6* | 62 ± n.r. | 0.61 ± 0.36 |
| Philis-Tsimikas et al. 2013 IIb (10) | 26 | Weekly | 1.10 ± 3.2* | 48 ± n.r. | 0.50 ± 0.29 |
| **All studies in this group** | **29 ± 9** |  | **1.3 ± 4.5** | **62 ± 36** | **0.66 ± 0.31** |
| **FPG Titration target 5.01-5.6 mmol/l** | |  |  |  |  |
| Aroda et al. 2017 (11) | 30 | Twice a week/ every 3 days | 1.2 ± 4.4 | 29 ± 16 | 0.31 ± 0.17 |
| Bolli et al. 2015 a (12) | 24 | Weekly | 0.5 ± 3.6 | 59 ± 32 | 0.62 ± 0.29 |
| Bolli et al. 2015 b (12) | 24 | Weekly | 0.7 ± 3.7 | 52 ± 28 | 0.53 ± 0.24 |
| D'Alessio et al. 2015 (13) | 24 | Twice a week/ every 3 days | 2.0 ± 4.0 | 52 ± 34 | 0.54 ± 0.31 |
| Davies et al. 2013 (14) | 26 | Weekly | 0.8 ± 3.7 | 51 ± 28 | 0.51 ± 0.30 |
| Diamant et al. 2010 (15) | 26 | Twice a week/ every 3 days | 1.4 ± 3.0 | 31 ± n.r. | 0.34 ± 0.20 |
| Fritsche et al. 2003 a (16) | 24 | During official study visits | 2.9 ± 4.3 | 37 ± 22 | 0.44 ± 0.26 |
| Fritsche et al. 2003 b (16) | 24 | During official study visits | 3.7 ± 3.6 | 39 ± 21 | 0.45 ± 0.24 |
| Fritsche et al. 2003 c (16) | 24 | During official study visits | 3.9 ± 4.5 | 40 ± 24 | 0.47 ± 0.28 |
| Heine et al. 2005 (17) | 26 | Twice a week/ every 3 days | 1.8 ± 4.9 | 25 ± n.r. | 0.28 ± 0.16 |
| Janka et al. 2005 (18) | 24 | Twice a week/ every 3 days | 1.4 ± 3.4 | 28 ± 15 | 0.33 ± 0.18 |
| Riddle et al. 2013 (19) | 24 | Weekly | 1.2 ± 4.5 | 50 ± 26 | 0.57 ± 0.30 |
| Weissman et al. 2014 (20) | 52 | Weekly | 1.6 ± 3.8 | 35 ± 26 | 0.36 ± 0.27 |
| Yki-Järvinen et al. 2006 (21) | 36 | Twice a week/ every 3 days | 2.6 ± 4.7 | 68 ± 39 | 0.69 ± 0.39 |
| Aschner et al. 2012 (22) | 24 | Twice a week/ every 3 days | 0.4 ± 3.3 | 41 ± 26 | 0.49 ± 0.36 |
| Riddle et al. 2003 a (23) | 24 | Weekly | 3.0 ± 3.8 | 47 ± n.r. | 0.48 ± 0.19 |
| Riddle et al. 2003 b (23) | 24 | Weekly | 2.8 ± 3.9 | 42 ± n.r. | 0.42 ± 0.20 |
| Blicklé et al. 2009 (24) | 36 | Twice a week/ every 3 days | 0.9 ± 2.9 | 23 ± 13 | 0.27 ± 0.15 |
| Home et al. 2015 a (25) | 36 | Weekly | 1.3 ± 3.0 | 32 ± 21 | 0.39 ± 0.22 |
| Home et al. 2015 b (25) | 36 | Weekly | 1.1 ± 3.0 | 31 ± 18 | 0.37 ± 0.19 |
| Russel-Jones et al. 2009 (26) | 26 | Twice a week/ every 3 days | 1.6 ± 5.0 | 24 ± n.r. | 0.28 ± 0.16 |
| Giorgino et al. 2015 (27) | 52 | Twice a week/ every 3 days | 1.4 ± 3.9 | 29 ± 26 | 0.33 ± 0.24 |
| Davies et al. 2009 (28) | 26 | Weekly | 3.0 ± 3.3 | 39 ± 24 | 0.38 ± 0.23 |
| Davies et al. 2016 a (29) | 52 | Weekly | 2.6 ± 9.8 | 38 ± 30 | 0.42 ± 0.32 |
| Davies et al. 2016 b (29) | 52 | Weekly | 2.1 ± 9.9 | 43 ± 27 | 0.45 ± 0.29 |
| Esposito et al. 2008 a (30) | 36 | Weekly | 2.4 ± 3.4 | 52 ± 16 | 0.61 ± 0.12 |
| Esposito et al. 2008 b (30) | 36 | Weekly | 2.8 ± 3.5 | 57 ± 15 | 0.67 ± 0.14 |
| Buse et al. 2009 (31) | 24 | Other | 2.5 ± 4.0 | 36 ± n.r. | 0.40 ± 0.23 |
| Standl et al. 2006 a (32) | 24 | During official study visits | 2.1 ± 6.0* | 35 ± 17 | n.r. |
| Standl et al. 2006 b (32) | 24 | During official study visits | 1.8 ± 5.2* | 32 ± 17 | n.r. |
| Rosenstock et al. 2016 I (33) | 30 | Weekly | 1.1 ± 4.3 | 40 ± 15 | 0.44 ± 0.16 |
| Strojek et al. 2010 a (34) | 24 | Weekly | 1.0 ± 3.5 | 33 ± 20 | 0.39 ± 0.24 |
| Strojek et al. 2010 b (34) | 24 | Weekly | 1.1 ± 3.3 | 30 ± 17 | 0.35 ± 0.20 |
| Roussel et al. 2019 a (35) | 30 | Twice a week/ every 3 days | 1.5 ± 3.4 | 53 ± 46 | 0.62 ± 0.54 |
| Roussel et al. 2019 b (35) | 30 | Twice a week/ every 3 days | 1.7 ± 3.9 | 61 ± 47 | 0.70 ± 0.54 |
| Vilsbøll et al. 2020 (36) | 52 | Twice a week/ every 3 days | 2.8 ± 5.5 | 38 ± n.r. | 0.41 ± 0.24 |
| Bretzel et al. 2008 (37) | 44 | Weekly | 3.0 ± 4.3 | 42 ± 26 | 0.49 ± 0.29 |
| Rosenstock et al. 2016 II (38) | 24 | n.r. | 0.4 ± 1.7 | 52 ± n.r. | 0.56 ± 0.33 |
| **All studies in this group** | **31 ± 10** |  | **1.9 ± 5.2** | **41 ± 25** | **0.46 ± 0.28** |
| **FPG Titration target ≥ 5.61 mmol/l** |  |  |  |  |  |
| Raskin et al. 2005 (39) | 28 | Weekly | 3.5 ± 4.5 | 51 ± 27 | 0.55 ± 0.27 |
| Fogelfeld et al. 2010 a (40) | 24 | Twice a week/ every 3 days | 1.9 ± 3.2 | 32 ± 19 | 0.39 ± 0.23 |
| Fogelfeld et al. 2010 b (40) | 24 | Twice a week/ every 3 days | 0.4 ± 2.9 | 37 ± 30 | 0.46 ± 0.36 |
| Nauck et al. 2013 (41) | 24 | Weekly | -0.4 ± 3.6 | 36 ±17 | 0.40 ± 0.19 |
| Rosenstock et al. 2008 a (42) | 52 | Weekly | 3.0 ± 6.8 | 71 ± n.r. | 0.78 ± 0.46 |
| Rosenstock et al. 2008 b (42) | 52 | Weekly | 3.9 ± 6.8 | 40 ± n.r. | 0.44 ± 0.26 |
| Hollander et. al. 2011 (43) | 26 | Weekly | -0.8 ± 2.3* | 54 ± n.r. | 0.59 ± 0.34 |
| **All studies in this group** | **33 ± 13** |  | **1.7 ± 4.9** | **47 ± 28** | **0.53 ± 0.31** |
| **All studies** | **31 ± 10** |  | **1.7 ± 5.0** | **48 ± 29** | **0.52 ± 0.31** |
| Continuous variables presented as means ± standard deviation, categorical variables are presented as number fulfilling/not  fulfilling the criterion and the proportion (percentage) fulfilling the criterion in question. Combined means and standard deviations for all studies belonging to one titration target category were calculated with standard equations; n.r.: not reported. *: Standard deviation has not been published and was approximated using the mean of all reported coefficients of variation. | | | | | |

**Supplementary Figure S 4.** Achievement of fasting plasma glucose titration targets in our systematic analysis of the influence of three categories of fasting plasma glucose titration targets on the success of basal insulin therapy introduced in insulin-naïve patients with type 2 diabetes treated with well-defined single or combined oral glucose-lowering agents. The proportion of subjects achieving their assigned fasting plasma glucose titration target was inferred from the mean ± standard deviation of end-of-study fasting plasma glucose concentrations assuming a normal distribution. The overall p-value was derived by χ^2^ test (contingency table analysis). Pairwise comparisons were derived from Fisher’s exact test.

| **Supplementary Table S6.** Fasting plasma glucose and HbA_1c_ concentrations as well as HbA_1c_ target achievement in clinical trials of basal insulin titration in insulin-naïve patients with type 2 diabetes using various basal insulin preparations in addition to well-defined single or combined oral glucose-lowering agents with different numbers of occasions for titrating basal insulin during the trials | | | | | |
| --- | --- | --- | --- | --- | --- |
|  | Outcome parameter | Fasting plasma glucose | HbA_1c_ | HbA_1c_ < 7% | HbA_1c_ ≤ 6.5% |
| Comparison | Unit | [mmol/] | [%] | yes/no (% yes) | yes/no (% yes) |
| Comparison across categories of titration occasions | Occasions of titration 1: 10-30 | 6.60 (6.56; 6.64) | 7.26 (7.24; 7.28) | 3208/3908 (45.1) | 1107/3614 (23.5) |
|  | Occasions of titration 2: 31-72 | 6.53 (6.48; 6.58) | 7.10 (7.08; 7.12) | 3693/3816 (49.2) | 688/1647 (29.5) |
|  | Overall significance (p-value) | 0.039 | < 0.0001 | < 0.0001 | < 0.0001 |
| Difference between categories of titration occasions | Δ between 10-30 vs. 31-72 occasions for titration | -0.07 (-0.00; -0.14) | -0.16 (-0.13; -0.19) | 4.1 (2.5; 5.7) | 6.0 (3.9; 8.2) |
| Continuous variables are presented mean and their 95%-confidence intervals, categorical variables are presented as number fulfilling/not fulfilling the criterion and the proportion (percentage) fulfilling the criterion in question. Statistical significance was assessed using one-way ANOVA (Welch’s test) for continuous variables and χ^2^ test for larger than 2 x 2 contingency tables and Fisher´s exact test for 2 x 2 contingency tables (e.g., *post hoc* tests to identify significant differences between specific titration occasions), including the “attributable difference” expressed as a percentage and its 95 % confidence interval. Exact p-values are presented. | | | | | |

| **Supplementary Table S7.** Insulin dose, hypoglycemia (any or severe) and body weight change from baseline in clinical trials of basal insulin titration in insulin-naïve patients with type 2 diabetes using various basal insulin preparations in addition to well-defined single or combined oral glucose-lowering agents with different numbers of occasions for titrating basal insulin during the trials | | | | | | |
| --- | --- | --- | --- | --- | --- | --- |
|  | Outcome parameter | Daily insulin dose | Daily insulin dose | Hypoglycemia | Severe hypoglycemia | Body weight change from baseline |
| Comparison | Unit | [U/d] | [U/kg/d] | yes/no (% yes) | yes/no (% yes) | [kg] |
| Comparison across categories of titration occasions | Occasions of titration 1: 10-30 | 45 (44; 45) | 0.48 (0.47; 0.49) | 3170/3833 (45.3) | 85/7840 (1.1) | 1.7 (1.6; 1.8) |
|  | Occasions of titration 2: 31-72 | 47 (47; 48) | 0.52 (0.51; 0.53) | 4321/2382 (64.5) | 78/7096 (1.1) | 1.7 (1.6; 1.8) |
|  | Overall significance (p-value) | < 0.0001 | < 0.0001 | < 0.0001 | ns | ns |
| Difference between categories of titration occasions | Δ between 10-30 vs. 31-72 occasions for titration | 2 (2; 4) | 0.04 (0.03; 0.05) | 19.2 (17.5; 20.9) | 0.0 (0.0; 0.0) | 0.0 (-0.2; 0.2) |
| Continuous variables are presented mean and their 95%-confidence intervals, categorical variables are presented as number fulfilling/not fulfilling the criterion and the proportion (percentage) fulfilling the criterion in question. Statistical significance was assessed using one-way ANOVA (Welch’s test) for continuous variables and χ^2^ test for larger than 2 x 2 contingency tables and Fisher´s exact test for 2 x 2 contingency tables (e.g., *post hoc* tests to identify significant differences between specific titration occasions), including the “attributable difference” expressed as a percentage and its 95 % confidence interval. Exact p-values are presented. | | | | | | |

| **Supplementary Table S8.** Fasting plasma glucose and HbA_1c_ concentrations as well as HbA_1c_ target achievement in clinical trials of basal insulin titration in insulin-naïve patients with type 2  diabetes using various basal insulin preparations in addition to well-defined single or combined oral glucose-lowering agents, using titration algorithms with varying “stringency” to address  hyperglycemia | | | | | |
| --- | --- | --- | --- | --- | --- |
|  | Outcome parameter | Fasting plasma glucose | HbA_1c_ | HbA_1c_ < 7% | HbA_1c_ ≤ 6.5% |
|  | Unit | [mmol/l] | [%] | yes/no (% yes) | yes/no (% yes) |
| Overall comparison across titration algorithms with varying degrees of stringency in addressing fasting hyperglycemia | Titration algorithm 1:  At investigator’s discretion | 6.16 (6.02; 6.30) | 7.04 (6.98; 7.10) | 750/658 (53.3) | 141/244 (36.6) |
|  | Titration algorithm 2:  One-step algorithm | 6.66 (6.55; 6.77) * | 7.14 (7.10; 7.18) * | 1188/1209 (49.6) * | 298/325 (47.8) * |
|  | Titration algorithm 3:  Stepped algorithm, weak (2-4 U) | 6.53 (6.48; 6.58) * | 7.09 (7.06; 7.12) | 1774/1833 (49.2) * | 458/1120 (29.0) *^, †^ |
|  | Titration algorithm 4:  Stepped algorithm, strong (≥ 5 U) | 6.61 (6.56; 6.65) * | 7.23 (7.21; 7.25) *^, †, ‡^ | 4255/4999 (46.0) *^, †, ‡^ | 1058/3806 (21.8 *^, †, ‡^ |
|  | Overall significance (p-value) | < 0.0001 | < 0.0001 | < 0.0001 | < 0.0001 |
| *Post hoc* comparison of all titration  algorithms | Δ titration algorithm  At investigator’s discretion vs.  one-step algorithm | 0.50 (0.27; 0.73) ^§^ | 0.10 (0.00; 0.20) ^§^ | -3.7 (-0.07; 0.00) ^§^ | 11.2 (4.9; 17.6;) ^§^ |
|  | Δ titration algorithm  At investigator’s discretion vs.  Stepped algorithm, weak (2-4 U) | 0.37 (0.17; 0.57) ^§^ | 0.05 (-0.04; 0.14) | -4.1 (-7.2; -1.0) ^§^ | -7.6 (-12.7; -2.5) ^§^ |
|  | Δ titration algorithm  At investigator’s discretion vs.  Stepped algorithm, strong (≥ 5 U) | 0.45 (0.26; 0.64) ^§^ | 0.19 (0.11; 0.27) ^§^ | -7.3 (-10.1; -4.5) ^§^ | -14.9 (-19.2; -10.5) ^§^ |
|  | Δ titration algorithm  One-step algorithm vs.  Stepped algorithm, weak (2-4 U) | -0.13 (-0.29; 0.03) | -0.05 (-0.12; 0.02) | -0.4 (-3.0; 2.2) | -18.8 (-23.2; -14.4) ^§^ |
|  | Δ titration algorithm  One-step algorithm vs.  Stepped algorithm, strong (≥ 5 U) | -0.05 (-0.20; 0.10) | 0.09 (0.03; 0.15) ^§^ | -3.6 (-5.8; -1.3) ^§^ | -26.1 (-30.0; -22.5) ^§^ |
|  | Δ titration algorithm  Stepped algorithm, weak (2-4 U) vs.  Stepped algorithm, strong (≥ 5 U) | 0.08 (-0.01; 0.17) | 0.14 (0.09; 0.19) ^§^ | -3.2 (-5.1; -1.3) ^§^ | -7.3 (-9.7; -4.9) ^§^ |
| Continuous variables are presented mean and their 95%-confidence intervals, categorical variables are presented as number fulfilling/not fulfilling the criterion and the proportion (percentage) fulfilling the criterion in question. Statistical significance was assessed using one-way ANOVA (Welch’s test) for continuous variables and χ^2^ test for larger than 2 x 2 contingency tables and Fisher´s exact test for 2 x 2 contingency tables (e.g., *post hoc* tests to identify significant differences between specific titration algorithms), including the “attributable difference” expressed as a percentage and its 95 % confidence interval. For overall comparisons, exact p-values are presented. *: Significantly different (p < 0.05) vs. titration algorithm 1 (at investigator´s discretion). ^†^: Significantly different (p < 0.05) vs. titration algorithm 2 (one-step algorithm). ^‡^: Significantly different (p < 0.05) vs. titration algorithm 3 (stepped algorithms, weak). For comparison of individual titration algorithms, significance is indicated by ^§^. | | | | | |

| **Supplementary Table S9.** Results achieved in clinical trials of basal insulin titration in insulin-naïve patients with type 2 diabetes using various basal insulin preparations in addition to well-defined single or combined  oral glucose-lowering agents, using different titration algorithms | | | | | | |
| --- | --- | --- | --- | --- | --- | --- |
|  | Outcome parameter | Daily insulin dose | Daily insulin dose | Hypoglycemia | Severe hypoglycemia | Body weight change  from baseline |
|  | Unit | [U/d] | [U/kg/d] | yes/no (% yes) | yes/no (% yes) | [kg] |
| Overall comparison across titration algorithms with varying degrees of stringency in addressing fasting hyperglycemia | Titration algorithm 1:  At investigator’s discretion | 54 (52; 55) | 0.59 (0.57; 0.61) | 442/966 (31.4) | 7/1401 (0.5) | 1.9 (1.7; 2.1) |
|  | Titration algorithm 2:  One-step algorithm | 58 (57; 60) * | 0.63 (0.62; 0.65) * | 1199/931 (56.3) *^,^ | 24/2163 (1.1) | 1.7 (1.5; 1.9) |
|  | Titration algorithm 3:  Stepped algorithm, weak (2-4 U) | 43 (42; 44) *^, †^ | 0.49 (0.48; 0.50) *^, †^ | 1679/1983 (45.8) *^, †^ | 40/3733 (1.1) | 1.3 (1.2; 1.4) *^, †^ |
|  | Titration algorithm 4:  Stepped algorithm, strong (≥ 5 U) | 43 (42; 43) *^, †^ | 0.47 (0.46; 0.48) *^, †, ‡^ | 4891/4306 (53.2) *^, †, ‡^ | 105/9667 (1.1) ^a^ | 1.9 (1.8; 2.0) ^‡^ |
|  | Overall significance (p-value) | < 0.0001 | < 0.0001 | < 0.0001 | 0.24 | < 0.0001 |
| *Post hoc* comparison of all titration  algorithms | Δ titration algorithm  At investigator’s discretion vs.  one-step algorithm | 4 (2; 7) ^§^ | 0.04 (0.01; 0.07) ^§^ | 24.9 (21.7; 28.2) ^§^ | 0.6 (0.0;0.0) | -0.2 (-0.7; 0.1) |
|  | Δ titration algorithm  At investigator’s discretion vs.  Stepped algorithm, weak (2-4 U) | -11 (-13; -8) ^§^ | -0.10 (-0.13; -0.07) ^§^ | 14.5 (11.6; 17.4) ^§^ | 0.6 (0.0;0.0) | -0.6 (-1.0; -0.3) ^§^ |
|  | Δ titration algorithm  At investigator’s discretion vs.  Stepped algorithm, strong (≥ 5 U) | -11 (-13; -9) ^§^ | -0.12 (-0.14; -0.10) ^§^ | 21.8 (19.2; 24.5) ^§^ | 0.6 (0.2; 1.2) ^§^ | -0.1 (-0.4; 0.3) |
|  | Δ titration algorithm  One-step algorithm vs.  Stepped algorithm, weak (2-4 U) | -15 (-17; -13) ^§^ | -0.14 (-0.16; -0.12) ^§^ | -10.5 (-13.1; -7.8) ^§^ | 0.0 (-0.7; 0.5) | -0.4 (-0.7; -0.1) ^§^ |
|  | Δ titration algorithm  One-step algorithm vs.  Stepped algorithm, strong (≥ 5 U) | -15 (-18; -14) ^§^ | -0.16 (-0.18; -0.14) ^§^ | -3.1 (-5.5; -0.7) ^§^ | 0.0 (-0.6; 0.4) | 0.2 (-0.1; 0.5) |
|  | Δ titration algorithm  Stepped algorithm, weak (2-4 U) vs.  Stepped algorithm, strong (≥ 5 U) | 0 (-2; 1) | -0.02 (-0.01; -0.04) ^§^ | 7.3 (5.4; 9.3) ^§^ | 0.0 (-0.5; 0.4) | 0.6 (0.4; 0.8) ^§^ |
| Continuous variables are presented mean and their 95%-confidence intervals, categorical variables are presented as number fulfilling/not fulfilling the criterion and the proportion (percentage) fulfilling the criterion in question. Statistical significance was assessed using one-way ANOVA (Welch’s test) for continuous variables and χ^2^ test for larger than 2 x 2 contingency tables and Fisher´s exact test for 2 x 2 contingency tables (e.g., *post hoc* tests to identify significant differences between specific titration algorithms), including the “attributable difference” expressed as a percentage and its 95 % confidence interval. For overall comparisons, exact p-values are presented. *: Significantly different (p < 0.05) vs. titration algorithm 1 (at investigator´s discretion). ^†^: Significantly different (p < 0.05) vs. titration algorithm 2 (one-step algorithm). ^‡^: Significantly different (p < 0.05) vs. titration algorithm 3 (stepped algorithms, weak). For comparison of individual titration algorithms, significance is indicated by ^§^. | | | | | | |

| **Supplementary Table S10**. Heterogeneity of fasting plasma glucose and HbA_1c_ after basal insulin titration with various glucose-lowering  medications used in insulin-native Patients with Type 2 diabetes aiming for different fasting plasma glucose titration targets in studies included in the present analysis | | | | |
| --- | --- | --- | --- | --- |
|  | Fasting plasma glucose |  | HbA_1c_ |  |
|  | Q | I² [%] | Q | I² [%] |
| FPG Titration target ≤ 5.0 mmol/l | 0.46 | 0 | 0.22 | 0 |
| FPG Titration target 5.01-5.6 mmol/l | 1.34 | 0 | 0.69 | 0 |
| FPG Titration target ≥ 5.61 mmol/l | 0.79 | 0 | 0.39 | 0 |
| All FPG TT categories | 1.39 | 0 | 0.47 | 0 |
| Heterogeneity was assessed using χ^2^ test (Q) and I². None of the Q values indicated a significant heterogeneity. Also, I^2^ < 35 % indicates negligible heterogeneity. | | | | |

**References**

1. Gough SC, Bode B, Woo V, Rodbard HW, Linjawi S, Poulsen P, Damgaard LH, Buse JB, investigators NNt. Efficacy and safety of a fixed-ratio combination of insulin degludec and liraglutide (IDegLira) compared with its components given alone: results of a phase 3, open-label, randomised, 26-week, treat-to-target trial in insulin-naive patients with type 2 diabetes. Lancet Diabetes Endocrinol 2014;2:885-893

2. Niswender K, Piletic M, Andersen H, Conradsen Hiort L, Hollander P. Weight change upon once-daily initiation of insulin detemir with or without dietary intervention in overweight or obese insulin-naive individuals with type 2 diabetes: results from the DIET trial. Diabetes Obes Metab 2014;16:186-192

3. Zinman B, DeVries JH, Bode B, Russell-Jones D, Leiter LA, Moses A, Johansen T, Ratner R, Nn, Investigators NNT. Efficacy and safety of insulin degludec three times a week versus insulin glargine once a day in insulin-naive patients with type 2 diabetes: results of two phase 3, 26 week, randomised, open-label, treat-to-target, non-inferiority trials. Lancet Diabetes Endocrinol 2013;1:123-131

4. Zinman B, Philis-Tsimikas A, Cariou B, Handelsman Y, Rodbard HW, Johansen T, Endahl L, Mathieu C, Investigators NNT. Insulin degludec versus insulin glargine in insulin-naive patients with type 2 diabetes: a 1-year, randomized, treat-to-target trial (BEGIN Once Long). Diabetes Care 2012;35:2464-2471

5. Gough SC, Bhargava A, Jain R, Mersebach H, Rasmussen S, Bergenstal RM. Low-volume insulin degludec 200 units/ml once daily improves glycemic control similarly to insulin glargine with a low risk of hypoglycemia in insulin-naive patients with type 2 diabetes: a 26-week, randomized, controlled, multinational, treat-to-target trial: the BEGIN LOW VOLUME trial. Diabetes Care 2013;36:2536-2542

6. Meneghini L, Kesavadev J, Demissie M, Nazeri A, Hollander P. Once-daily initiation of basal insulin as add-on to metformin: a 26-week, randomized, treat-to-target trial comparing insulin detemir with insulin glargine in patients with type 2 diabetes. Diabetes Obes Metab 2013;15:729-736

7. Philis-Tsimikas A, Billings LK, Busch R, Portillo CM, Sahay R, Halladin N, Eggert S, Begtrup K, Harris S. Superior efficacy of insulin degludec/liraglutide versus insulin glargine U100 as add-on to sodium-glucose co-transporter-2 inhibitor therapy: A randomized clinical trial in people with uncontrolled type 2 diabetes. Diabetes Obes Metab 2019;21:1399-1408

8. Philis-Tsimikas A, Del Prato S, Satman I, Bhargava A, Dharmalingam M, Skjoth TV, Rasmussen S, Garber AJ. Effect of insulin degludec versus sitagliptin in patients with type 2 diabetes uncontrolled on oral antidiabetic agents. Diabetes Obes Metab 2013;15:760-766

9. Kumar A, Franek E, Wise J, Niemeyer M, Mersebach H, Simo R. Efficacy and safety of once-daily insulin Degludec/insulin Aspart versus insulin Glargine (U100) for 52 Weeks in insulin-naive patients with type 2 diabetes: A randomized controlled trial. PLoS One 2016;11:e0163350

10. Philis-Tsimikas A, Brod M, Niemeyer M, Ocampo Francisco AM, Rothman J. Insulin degludec once-daily in type 2 diabetes: simple or step-wise titration (BEGIN: once simple use). Adv Ther 2013;30:607-622

11. Aroda VR, Bain SC, Cariou B, Piletic M, Rose L, Axelsen M, Rowe E, DeVries JH. Efficacy and safety of once-weekly semaglutide versus once-daily insulin glargine as add-on to metformin (with or without sulfonylureas) in insulin-naive patients with type 2 diabetes (SUSTAIN 4): a randomised, open-label, parallel-group, multicentre, multinational, phase 3a trial. Lancet Diabetes Endocrinol 2017;5:355-366

12. Bolli GB, Riddle MC, Bergenstal RM, Ziemen M, Sestakauskas K, Goyeau H, Home PD, on behalf of the Esi. New insulin Glargine 300 U/ml compared with Glargine 100 U/ml in insulin-naive people with type 2 diabetes on oral glucose-lowering drugs: A randomized controlled trial (EDITION 3). Diabetes Obes Metab 2015;17:386-394

13. D'Alessio D, Haring HU, Charbonnel B, de Pablos-Velasco P, Candelas C, Dain MP, Vincent M, Pilorget V, Yki-Jarvinen H, Investigators E. Comparison of insulin glargine and liraglutide added to oral agents in patients with poorly controlled type 2 diabetes. Diabetes Obes Metab 2015;17:170-178

14. Davies M, Heller S, Sreenan S, Sapin H, Adetunji O, Tahbaz A, Vora J. Once-weekly exenatide versus once- or twice-daily insulin Detemir: Randomized, open-label, clinical trial of efficacy and safety in patients with type 2 diabetes treated with metformin alone or in combination with sulfonylureas. Diabetes Care 2013;36:1368-1376

15. Diamant M, Van Gaal L, Stranks S, Northrup J, Cao D, Taylor K, Trautmann M. Once weekly Exenatide compared with insulin Glargine titrated to target in patients with type 2 diabetes (DURATION-3): An open-label randomised trial. Lancet 2010;375:2234-2243

16. Fritsche A, Schweitzer MA, Haring HU, Study G. Glimepiride combined with morning insulin glargine, bedtime neutral protamine hagedorn insulin, or bedtime insulin glargine in patients with type 2 diabetes. A randomized, controlled trial. Ann Intern Med 2003;138:952-959

17. Heine RJ, Van Gaal LF, Johns D, Mihm MJ, Widel MH, Brodows RG, Group GS. Exenatide versus insulin Glargine in patients with suboptimally controlled type 2 diabetes: A randomized trial. Ann Intern Med 2005;143:559-569

18. Janka HU, Plewe G, Riddle MC, Kliebe-Frisch C, Schweitzer MA, Yki-Jarvinen H. Comparison of basal insulin added to oral agents versus twice-daily premixed insulin as initial insulin therapy for type 2 diabetes. Diabetes Care 2005;28:254-259

19. Riddle MC, Forst T, Aronson R, Sauque-Reyna L, Souhami E, Silvestre L, Ping L, Rosenstock J. Adding once-daily Lixisenatide for type 2 diabetes inadequately controlled with newly initiated and continuously titrated basal insulin Glargine: A 24-week, randomized, placebo-controlled study (GetGoal-Duo 1). Diabetes Care 2013;36:2497-2503

20. Weissman PN, Carr MC, Ye J, Cirkel DT, Stewart M, Perry C, Pratley R. HARMONY 4: Randomised clinical trial comparing once-weekly Albiglutide and insulin Glargine in patients with type 2 diabetes inadequately controlled with metformin with or without sulfonylurea. Diabetologia 2014;57:2475-2584

21. Yki-Järvinen H, Kauppinen-Makelin R, Tiikkainen M, Vahatalo M, Virtamo H, Nikkila K, Tulokas T, Hulme S, Hardy K, McNulty S, Hanninen J, Levanen H, Lahdenpera S, Lehtonen R, Ryysy L. Insulin glargine or NPH combined with metformin in type 2 diabetes: the LANMET study. Diabetologia 2006;49:442-451

22. Aschner P, Chan J, Owens DR, Picard S, Wang E, Dain MP, Pilorget V, Echtay A, Fonseca V, investigators E. Insulin Glargine versus Sitagliptin in insulin-naive patients with type 2 diabetes mellitus uncontrolled on metformin (EASIE): A multicentre, randomised open-label trial. Lancet 2012;379:2262-2269

23. Riddle MC, Rosenstock J, Gerich J, Insulin Glargine Study I. The treat-to-target trial: Randomized addition of Glargine or human NPH insulin to oral therapy of type 2 diabetic patients. Diabetes Care 2003;26:3080-3086

24. Blickle JF, Hancu N, Piletic M, Profozic V, Shestakova M, Dain MP, Jacqueminet S, Grimaldi A. Insulin Glargine provides greater improvements in glycaemic control vs. intensifying lifestyle management for people with type 2 diabetes treated with OADs and 7-8% A1c levels. The TULIP study. Diabetes Obes Metab 2009;11:379-386

25. Home PD, Bolli GB, Mathieu C, Deerochanawong C, Landgraf W, Candelas C, Pilorget V, Dain MP, Riddle MC. Modulation of insulin dose titration using a hypoglycaemia-sensitive algorithm: Insulin Glargine versus neutral protamine Hagedorn insulin in insulin-naive people with type 2 diabetes. Diabetes Obes Metab 2015;17:15-22

26. Russell-Jones D, Vaag A, Schmitz O, Sethi BK, Lalic N, Antic S, Zdravkovic M, Ravn GM, Simo R, Liraglutide E, Action in Diabetes 5 met SUSG. Liraglutide vs insulin Glargine and placebo in combination with metformin and sulfonylurea therapy in type 2 diabetes mellitus (LEAD-5 met+SU): A randomised controlled trial. Diabetologia 2009;52:2046-2055

27. Giorgino F, Benroubi M, Sun JH, Zimmermann AG, Pechtner V. Efficacy and safety of once-weekly Dulaglutide versus insulin Glargine in patients with zype 2 diabetes on Metformin and Glimepiride (AWARD-2). Diabetes Care 2015;38:2241-2249

28. Davies MJ, Donnelly R, Barnett AH, Jones S, Nicolay C, Kilcoyne A. Exenatide compared with long-acting insulin to achieve glycaemic control with minimal weight gain in patients with type 2 diabetes: results of the Helping Evaluate Exenatide in patients with diabetes compared with Long-Acting insulin (HEELA) study. Diabetes Obes Metab 2009;11:1153-1162

29. Davies MJ, Russell-Jones D, Selam JL, Bailey TS, Kerenyi Z, Luo J, Bue-Valleskey J, Ivanyi T, Hartman ML, Jacobson JG, Jacober SJ, Investigators IS. Basal insulin Peglispro versus insulin Glargine in insulin-naive type 2 diabetes: IMAGINE 2 randomized trial. Diabetes Obes Metab 2016;18:1055-1064

30. Esposito K, Ciotola M, Maiorino MI, Gualdiero R, Schisano B, Ceriello A, Beneduce F, Feola G, Giugliano D. Addition of neutral protamine lispro insulin or insulin glargine to oral type 2 diabetes regimens for patients with suboptimal glycemic control: A randomized trial. Ann Intern Med 2008;149:531-519

31. Buse JB, Wolffenbuttel BH, Herman WH, Shemonsky NK, Jiang HH, Fahrbach JL, Scism-Bacon JL, Martin SA. DURAbility of basal versus lispro mix 75/25 insulin efficacy (DURABLE) trial 24-week results: Safety and efficacy of insulin lispro mix 75/25 versus insulin Glargine added to oral antihyperglycemic drugs in patients with type 2 diabetes. Diabetes Care 2009;32:1007-1013

32. Standl E, Maxeiner S, Raptis S, Group HOES. Once-daily insulin Glargine administration in the morning compared to bedtime in combination with morning Glimepiride in patients with type 2 diabetes: An assessment of treatment flexibility. Horm Metab Res 2006;38:172-177

33. Rosenstock J, Aronson R, Grunberger G, Hanefeld M, Piatti P, Serusclat P, Cheng X, Zhou T, Niemoeller E, Souhami E, Davies M, LixiLan OTI. Benefits of LixiLan, a titratable fixed-ratio combination of insulin Glargine plus Lixisenatide, versus insulin Glargine and lixisenatide monocomponents in Type 2 diabetes inadequately controlled on oral agents: The LixiLan-O randomized trial. Diabetes Care 2016;39:2026-2035

34. Strojek K, Shi C, Carey MA, Jacober SJ. Addition of insulin lispro protamine suspension or insulin Glargine to oral type 2 diabetes regimens: A randomized trial. Diabetes Obes Metab 2010;12:916-922

35. Roussel R, Duran-Garcia S, Zhang Y, Shah S, Darmiento C, Shankar RR, Golm GT, Lam RLH, O'Neill EA, Gantz I, Kaufman KD, Engel SS. Double-blind, randomized clinical trial comparing the efficacy and safety of continuing or discontinuing the dipeptidyl peptidase-4 inhibitor Sitagliptin when initiating insulin Glargine therapy in patients with type 2 diabetes: The CompoSIT-I Study. Diabetes Obes Metab 2019;21:781-790

36. Vilsboll T, Ekholm E, Johnsson E, Garcia-Sanchez R, Dronamraju N, Jabbour SA, Lind M. Efficacy and safety of dapagliflozin plus saxagliptin versus insulin glargine over 52 weeks as add-on to metformin with or without sulphonylurea in patients with type 2 diabetes: A randomized, parallel-design, open-label, Phase 3 trial. Diabetes Obes Metab 2020;22:957-968

37. Bretzel RG, Nuber U, Landgraf W, Owens DR, Bradley C, Linn T. Once-daily basal insulin Glargine versus thrice-daily prandial insulin Lispro in people with type 2 diabetes on oral hypoglycaemic agents (APOLLO): An open randomised controlled trial. Lancet 2008;371:1073-1084

38. Rosenstock J, Diamant M, Aroda VR, Silvestre L, Souhami E, Zhou T, Perfetti R, Fonseca V, LixiLan Po CSG. Efficacy and Safety of LixiLan, a Titratable Fixed-Ratio Combination of Lixisenatide and Insulin Glargine, Versus Insulin Glargine in Type 2 Diabetes Inadequately Controlled on Metformin Monotherapy: The LixiLan Proof-of-Concept Randomized Trial. Diabetes Care 2016;39:1579-1586

39. Raskin P, Allen E, Hollander P, Lewin A, Gabbay RA, Hu P, Bode B, Garber A, Group IS. Initiating insulin therapy in type 2 diabetes: A comparison of biphasic and basal insulin analogs. Diabetes Care 2005;28:260-265

40. Fogelfeld L, Dharmalingam M, Robling K, Jones C, Swanson D, Jacober S. A randomized, treat-to-target trial comparing insulin lispro protamine suspension and insulin Detemir in insulin-naive patients with type 2 diabetes. Diabet Med 2010;27:181-188

41. Nauck M, Horton E, Andjelkovic M, Ampudia-Blasco FJ, Parusel CT, Boldrin M, Balena R, Group TeS. Taspoglutide, a once-weekly glucagon-like peptide 1 analogue, vs. insulin Glargine titrated to target in patients with type 2 diabetes: An open-label randomized trial. Diabet Med 2013;30:109-113

42. Rosenstock J, Davies M, Home PD, Larsen J, Koenen C, Schernthaner G. A randomised, 52-week, treat-to-target trial comparing insulin detemir with insulin glargine when administered as add-on to glucose-lowering drugs in insulin-naive people with type 2 diabetes. Diabetologia 2008;51:408-416

43. Hollander P, Raslova K, Skjoth TV, Rastam J, Liutkus JF. Efficacy and safety of insulin Detemir once daily in combination with Sitagliptin and metformin: The TRANSITION randomized controlled trial. Diabetes Obes Metab 2011;13:268-275
